# Efficient expansion of rare human circulating hematopoietic stem/progenitor cells in steady-state blood using a polypeptide-forming 3D culture

**DOI:** 10.1007/s13238-021-00900-4

**Published:** 2022-03-01

**Authors:** Yulin Xu, Xiangjun Zeng, Mingming Zhang, Binsheng Wang, Xin Guo, Wei Shan, Shuyang Cai, Qian Luo, Honghu Li, Xia Li, Xue Li, Hao Zhang, Limengmeng Wang, Yu Lin, Lizhen Liu, Yanwei Li, Meng Zhang, Xiaohong Yu, Pengxu Qian, He Huang

**Affiliations:** 1 Bone Marrow Transplantation Center, First Affiliated Hospital, Zhejiang University School of Medicine, Hangzhou, 310012 China; 2 Liangzhu Laboratory, Zhejiang University Medical Center, Hangzhou, 311121 China; 3 Institute of Hematology, Zhejiang University, Hangzhou, 310012 China; 4 Zhejiang Province Engineering Laboratory for Stem Cell and Immunity Therapy, Hangzhou, 310012 China; 5 Core Facilities, Zhejiang University School of Medicine, Hangzhou, 310012 China; 6 Dr. Li Dak Sum & Yip Yio Chin Center for Stem Cell and Regenerative Medicine, Zhejiang University, Hangzhou, 310012 China

**Keywords:** hematopoietic stem/progenitor cell transplantation, peripheral blood mononuclear cells, expansion, mobilization, three-dimensional culture, self-renewal and multilineage differentiation

## Abstract

Although widely applied in treating hematopoietic malignancies, transplantation of hematopoietic stem/progenitor cells (HSPCs) is impeded by HSPC shortage. Whether circulating HSPCs (cHSPCs) in steady-state blood could be used as an alternative source remains largely elusive. Here we develop a three-dimensional culture system (3DCS) including arginine, glycine, aspartate, and a series of factors. Fourteen-day culture of peripheral blood mononuclear cells (PBMNCs) in 3DCS led to 125- and 70-fold increase of the frequency and number of CD34^+^ cells. Further, 3DCS-expanded cHSPCs exhibited the similar reconstitution rate compared to CD34^+^ HSPCs in bone marrow. Mechanistically, 3DCS fabricated an immunomodulatory niche, secreting cytokines as TNF to support cHSPC survival and proliferation. Finally, 3DCS could also promote the expansion of cHSPCs in patients who failed in HSPC mobilization. Our 3DCS successfully expands rare cHSPCs, providing an alternative source for the HSPC therapy, particularly for the patients/donors who have failed in HSPC mobilization.

**Supplementary Information:**

The online version contains supplementary material available at 10.1007/s13238-021-00900-4.

## INTRODUCTION

The majority of hematopoietic cells circulating in the peripheral blood (PB) are mature lineage cells, which are regularly replenished by hematopoietic stem and progenitor cells (HSPCs) (Massberg et al., 2007). To maintain a steady stem cell pool and to produce downstream progenies, HSPCs possess extensive self-renewal and multilineage differentiation capacities, which balance the requirement for various cell types in the circulation each day (Boulais and Frenette, 2015; Calvi and Link, 2015).

Studies have shown that quiescent HSPCs are a very rare population (<1 in 50,000 mononuclear cells) and predominantly reside in bone marrow (BM) (Mayani et al., 2003; Haylock et al., 2007; Wisniewski et al., 2011; Wei and Frenette, 2018). Due to the stimulation of SDF-1-CXCR4 signaling pathway, a few HSPCs migrate between PB and BM niches, and the rare migratory HSPCs circulating in PB is defined as circulating hematopoietic stem/progenitor cells (cHSPCs) (Sonoda, 2008; Massberg and von Andrian, 2009). Decades of effort have led to successful transplantation of HSPCs (HSPCT) for the treatment of hematopoietic malignancies and immune system disorders (Mimeault et al., 2007; Luo et al., 2014). However, there are very few HSPCs circulating in normal PB (Mayani et al., 2003; McKinney-Freeman and Goodell, 2004; Schreier and Triampo, 2020). To harvest enough HSPCs for transplantation, mobilization reagents such as granulocyte-colony stimulating factor (G-CSF) or CXCR4 antagonist plerixafor are necessarily administered to donors to mobilize HSPC from BM (Hequet, 2015). Unfortunately, approximately 5%–46% of donors still fail to show HSPC mobilization (Bensinger et al., 2009; Perseghin et al., 2009; Forristal et al., 2015).

To obtain a sufficient amount of HSPCs for clinical HSPCT, many efforts have been made for the *ex vivo* expansion of HSPC population or induction of pluripotent stem cells into HSPC-like cells (Bodine et al., 1989; Ku et al., 1996; Reya et al., 2003; De Angeli et al., 2004; Boitano et al., 2010; Xu et al., 2016; Bai et al., 2019; Wilkinson et al., 2019b; Shan et al., 2020). Currently, umbilical cord blood (UCB) and BM are employed as sources of HSPCs for *ex vivo* expansion (Boitano et al., 2010; Bai et al., 2019; Wilkinson et al., 2019b). Although collected in a simple and noninvasive manner, whether steady-state PB samples could be used as an alternative and efficient source for HSPCT remains largely unknown.

In this study, we developed a *de novo* three-dimensional culture system (3DCS), in which peripheral blood mononuclear cells (PBMNCs) isolated from PB without mobilization were seeded in polypeptide hydrogel self-assembled by amino acids of arginine, glycine, and aspartate (RGD), and cultured in hematopoietic medium supplemented with stem cell factor (SCF), flt3 ligand (FLT3L), thrombopoietin (TPO), interleukin-6 (IL-6), interleukin-3 (IL-3), vascular endothelial growth factor (VEGF) , StemRegenin1 (SR1) and Vitamin C (Vc). Our results showed that 3DCS led to robust expansion of phenotypic cHSPCs including CD34^+^ cells, and LIN^−^CD45RA^−^CD34^+^CD38^low/−^CD49f^+^CD90^+^ cells. Further, the 3DCS-expanded HSPCs maintained both long-term self-renewal and multilineage differentiation potentials via serial *in vitro* and *in vivo* assays. RNA-Seq elucidated the mechanisms underlying cHSPC expansion, by which the 3DCS fabricated an immunomodulatory microenvironment and provided cytokines as TNF-α to support HSPC survival and proliferation. Finally, we validated that 3DCS could also efficiently expand cHSPCs in patients who failed in BM-HSPC mobilization.

## RESULTS

### Optimization of 3DCS shows that the combination of VEGF, SR1, Vc plus 5 factors most significantly expanded cHSPCs

We initially optimized 3DCS by testing different combinations of growth factors and cytokines. Compared with the control group (StemSpan SFEM II plus 5 hematopoietic growth factors SCF, FLT3L, TPO, IL-3 and IL-6, defined as 5 factors), the addition of VEGF, SR1, Vc alone significantly increased the percentages and/or absolute numbers of LIN^−^CD45RA^−^CD34^+^CD38^low/−^CD49f^+^, and/or LIN^−^CD45RA^−^CD34^+^CD38^low/−^CD90^+^ and/or LIN^−^CD45RA^−^CD34^+^CD38^low/−^CD49f^+^CD90^+^ HSPC populations, and the combination of three small molecules showed the best efficiency in expanding cHSPCs (Fig. S1). Thus, we chose the basic HSPC medium supplemented with VEGF, SR1 and Vc as the culture recipe for the following study.

### 3DCS promotes the colony formation and proliferation of cHSPCs

Base on the optimal cocktails for cHSPC expansion, we investigated the effect of seeding density on the expansion of PBMNCs in 3DCS, and observed that the system was efficient to capture and proliferate rare cHSPCs even at low cell density of 1~2 × 10^6^ PBMNCs per well using 6 well plates. A higher seeding density as 4~5 × 10^6^ PBMNCs per well clearly compromised viability of the expanded cells. To fully utilize 3DCS capacity, we selected 1~2 × 10^6^ PBMNC density per well of 6-well plates for the further study. Therefore, in the study, we applied the culture condition described above for the further exploration.

**Figure 1:**
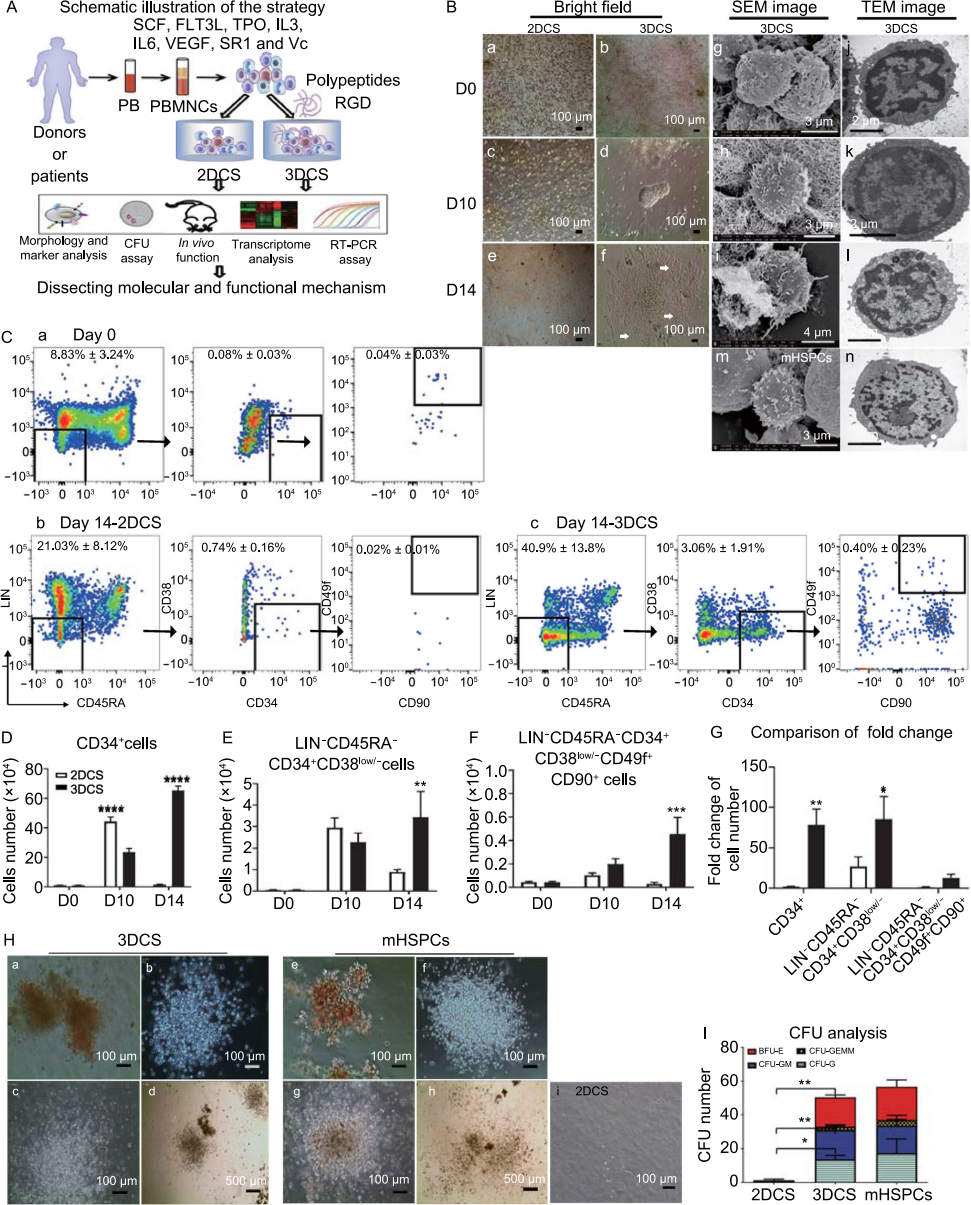
**3DCS facilitated the proliferation of rare cells circulating in normal peripheral blood**. (A) Scheme of the study. (B) The morphology change of 3DCS. a–f, Kinetic comparison of the morphological change in 2DCS and 3DCS. g–l, Kinetic cellular ultrastructure of the cells in 3DCS determined through SEM and TEM analysis. m and n are positive images from CD34^+^ HSPCs mobilized separately. (C) Typical flow cytometry plots were demonstrated to indicate the kinetics of cHSPC subpopulations in 2DCS (b) and 3DCS (c) compared to the cells at day 0 (a). (D–F) Kinetic analysis for cell number of cHSPC subsets. (G) Fold change comparison for cell numbers in 2DCS and 3DCS. (H) CFU-forming potential analysis for CD34^+^ cells in 3DCS (a–d), mHSPCs (e–h), and the samples in 2DCS (i). (I) Distribution of CFU subtypes. mHSPCs, mobilized CD34^+^ HSPCs. TNCs, total nucleated cells. Data are the means ± SEM from three or four replicates. *P < 0.05, **P < 0.01, ***P < 0.001, ****P < 0.0001

As shown in Fig. 1A, we examined the roles of 3DCS in cHSPC expansion. During the culture, 3DCS gradually produced cobble stone-like or vascular-like colonies, while no such colonies were formed in two-dimension culture system (2DCS) (Figs. 1B a-f, and S2A a-f). Next, we detected the ultrastructure of the cells in 3DCS by scanning electron microscopy (SEM) and transmission electron microscopy (TEM), and found that stem-like cells embedded in the polypeptide niche showed a high nuclear-cytoplasm ratio, similar to those of mobilized CD34^+^ HSPCs from BM (mHSPCs) (Fig. 1B g-n). We further measured the cell proliferation rate using the expression of Ki67 at the indicated time points, and found that 3DCS facilitated cell proliferation compared to 2DCS (Fig. S2B). Together, these results revealed that 3DCS promoted the colony formation and proliferation of the cells in steady-state PBMNCs.

### 3DCS preserves the primitive HSPC population

The kinetic changes of HSPC subpopulations were analyzed (Figs. 1C–G and S2C–E). The results showed that the frequency and count of CD34^+^ cells at day 14 in 3DCS increased by 125- and 70-fold respectively compared with day 0, and increased by 43.7- and 59.5-fold compared to 2DCS. Previous studies have documented that the HSPC population is enriched in the LIN^−^CD45RA^−^CD34^+^CD38^low/−^ population (Wisniewski et al., 2011; Ferreira et al., 2012). In our study, we observed a 122.7-fold increase in the frequency and a 76.4-fold increase in the absolute number of this subpopulation after 14-day culture in 3DCS compared with those at day 0, and these values represented a 4.13-fold and a 3.85-fold increase compared with 2DCS. Moreover, the frequency and absolute number of human primitive HSCs (LIN^−^CD45RA^−^CD34^+^CD38^low/−^CD49f^+^CD90^+^) (Majeti et al., 2007; Notta et al., 2011) were increased by 16- and 10.6-fold, respectively at day 14, in 3DCS compared with those at day 0, and by 17.8- and 15.1-fold in 3DCS compared to 2DCS.

Based on these data, we evaluated whether our 3DCS could expand enough HSPCs for transplantation given that a patient has body weight (bw) of 75 kg. Considering that at least 2 × 10^6^/kg/bw CD34^+^ HSPCs has been accepted as the threshold for successful hematopoietic recovery (Perseghin et al., 2009), 1.5 × 10^8^ CD34^+^ cells are required. According to the data from this study, starting with PBMNCs from 1 mL peripheral blood, ~6.3 × 10^5^ CD34^+^ cells were obtained after 14 days of 3DCS. Therefore, ~212 mL of PB was needed to obtain 1.5 × 10^8^ CD34^+^ cells, which is acceptable from a regular blood donation and sufficient for transplantation.

Collectively, these data suggest that the polypeptide hydrogel of 3DCS remarkably expanded the primitive cHSPCs in the normal peripheral blood without mobilization.

### 3DCS-expanded cHSPCs exhibit compelling *in vitro* and *in vivo* repopulating capacities

We then evaluated whether cHSPCs derived from 3DCS at day 14 held stem repopulating capacities. Colony-forming unit (CFU) assay showed that the 3DCS-derived cHSPCs markedly increased in the numbers of CFUs, including burst-forming unit-erythroids (BFU-Es), CFU-granulocytes (CFU-Gs), CFU-granulocytes/macrophages (CFU-GM) and CFU-granulocytes/erythrocytes/macrophages/megakaryocytes (CFU-GEMM) (Fig. 1H a-d), which were similar to the colonies formed by mHSPCs (Fig. 1H e-h), however, 2DCS-derived cells held poor potential to form CFUs after a 14-day culture (Fig. 1H i). The data were statistically shown in Fig. 1I.

**Figure 2:**
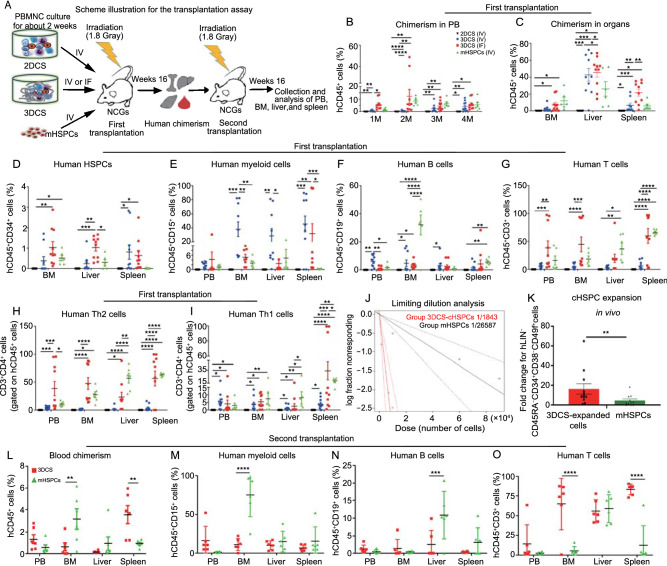
**3DCS-derived cHSPCs exhibited compelling** ***in vivo*** **repopulating capacities**. (A) Schematic diagram of the long-term engraftment capacity. (B–I) The results for the first transplantation. Human CD45^+^ cells were detected in PB after transplantation for 1 month, 2 months, 3 months and 4 months in the recipients (B) and in BM, livers and spleens after transplantation for 4M (C). The distribution of the human CD34^+^CD45^+^ HSPCs was verified in the hematopoietic organs of the recipients (D). The distribution of the human hematopoietic lineage was verified in the hematopoietic organs of the recipients (E–I). (J) Linear regression analysis for the limited dilution transplantation assay. Solid lines indicate the best-fit linear regression model for each dose set; dotted lines represent the 90% confidence intervals (Poisson statistic calculation). (K) cHSPC expansion potential assay for LIN^−^CD45RA^−^CD38^+^CD49f^+^ 3DCS-derived cells *in vivo*. (L–O) The results for the second transplantation. Data are presented as the means ± SEM from six or ten replicates. M, month. *P < 0.05, **P < 0.01, ***P < 0.001, ****P < 0.0001

**Figure 3:**
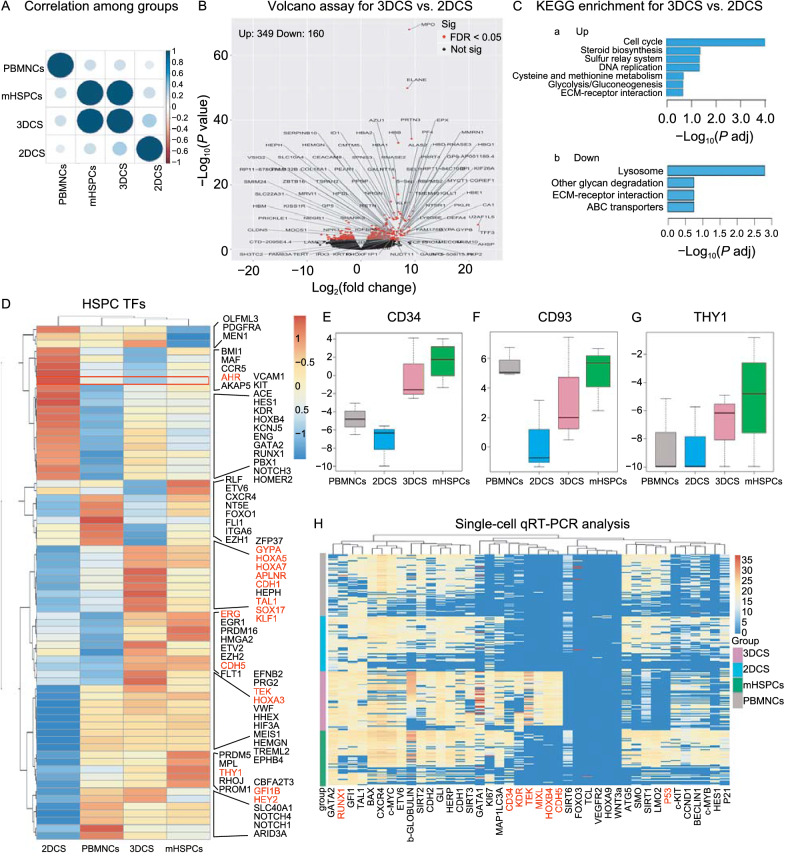
**Gene expression profiling analysis revealed the HSPC signatures of 3DCS-expanded cHSPCs**. (A) PCA revealed that the cells in 3DCS presented a similar expression pattern to mHSPCs. (B) Volcano analysis for 3DCS versus 2DCS. Some genes were labelled with the cutoff of |log2foldchange| > 5 and *P*-adj < 0.05. (C) Typical enrichment analysis of KEGG for the up- (a) and downregulated- (b) DEGs identified between 3DCS and 2DCS. (D–G) cHSPCs expressed HSPC-specific hematopoietic cell factors. (H) High-throughput qPCR analysis verified key hematopoietic transcription factor expression in cHSPCs with a heatmap. PCA, principal component analysis. KEGG, Kyoto Encyclopedia of Genes and Genomes

To test the *in vivo* repopulating potential, 3DCS-derived cells were intravenously (IV) or intrafemorally (IF) injected into immunocompromised mice (Fig. 2A). Strikingly, 3DCS-derived cells showed similar human chimerism as mHSPCs, while 2DCS-derived cells exhibited very low chimerism (Fig. 2B). IF injection produced higher chimerism than IV injection, indicating that BM is the major niche for human HSC reconstitution. Sixteen weeks after transplantation, the hematopoietic organs of the recipients were detected to have human chimerism including HSPCs, myeloid, B, and T cells similar to that of mHSPCs (Fig. 2C–I). Typical flow cytometry plots were shown in S Fig. 3. Poisson distribution analysis revealed an upper SRC frequency of 1/26,587 in LIN^−^CD45RA^−^CD34^+^CD38^low/−^ mHSPCs, and 1/1,843 in 3DCS-expanded counterparts using Extreme Limiting Dilution Analysis (Fig. 2J) (https://bioinf.wehi.edu.au/software/elda/) (Hu and Smyth, 2009), demonstrating that 3DCS-derived cells have a significant expansion of SRC numbers (Fig. 2J and Table S1). Furthermore, 3DCS-derived primitive LIN^−^CD45RA^−^CD34^+^CD38^low/−^CD49f^+^ HSPCs held greater amplification potential than mHSPCs did in the transplanted mice, and the expansion-fold change was 16.2 ± 5.26 versus 4.49 ± 1.68 respectively (Fig. 2K).

Secondary transplantation assays showed the human chimerism in BM, livers, and spleens as mHSPCs did (Fig. 2L). Besides, the presence of human hematopoietic lineages, such as myeloid cells, B cells, and T cells, in the recipients revealed the long-term engraftment capacity of 3DCS-derived cHSPCs (Fig. 2M–O). Typical flow cytometry plots were shown in Fig. S4.

Together, these data suggest that 3DCS-expanded HSPCs exhibit remarkable *in vitro* and *in vivo* repopulating capacities.

### Transcriptome analysis reveals that 3DCS-expanded cHSPCs are highly enriched with stemness programs

To demonstrate the molecular mechanisms underlying cHSPC, we executed RNA-seq for PBMNCs, 2DCS-derived cells, cHSPCs and mHSPCs. Principal component analysis (PCA) analysis revealed that cHSPC exhibited a similar transcriptome pattern as mHSPCs (Fig. 3A). A Venn diagram showed the number of differentially expressed genes (DEGs; |log2(fold change) | > 2 and adjusted *P* value (*P adj*) < 0.05) among the four groups (Fig. S5A).

By comparing 3DCS with 2DCS, we identified 349 upregulated and 160 downregulated genes (Fig. 3B). Kyoto Encyclopedia of Genes and Genomes (KEGG) and Gene Ontology (GO) analyses indicated that genes upregulated in cHSPCs were highly enriched in categories as the cell cycle and DNA replication (Figs. 3C, S5B, S5C, and Table S2). Gene set enrichment analysis (GSEA) revealed that signaling pathways essential for cell growth, such as epidermal growth factor receptor (EGFR) signaling, RNA binding, and DNA metabolic processes were enriched in cHSPCs (Fig. S5D a-e), whereas the pathways as STAT3 and lysosome clearance were upregulated in 2DCS (Fig. S5D f–j).

By comparison cHSPCs with mHSPCs, we found that 298 upregulated and 163 downregulated genes (Fig. S6A). KEGG analyses validated the similarity between cHSPCs and mHSPCs (Fig. S6B, S6C, and Table S3). Besides, GSEA revealed that mitochondrial metabolism and mRNA processing pathways were highly enriched in cHSPCs (Fig. S6D a–k). cHSPCs expressed much higher levels of HSPC-specific transcription factors (TFs) (Fig. 3D) and surface markers (Figs. 3E–G and S7). Intriguingly, we found that TFs essential for HSC self-renewal (*GFI1B*, *HOXA5*, *HOXA7*, and *TAL1*) and surface marker genes (*CD34*, *THY1*, *TIE2* and *CDH5*) were all enriched in cHSPCs. Additionally, the aryl hydrocarbon receptor (*AhR*), was expressed at a much lower level in cHSPCs, indicating the molecular mechanism involved in cHSPCs. Furthermore, cHSPCs expressed a higher level of markers related to mesoderm as *KDR*, *CDH5*, *CDH1*, *HEY2*, *APLENR*, and *SOX17*, suggesting that cHSPCs might share the similar molecular signatures with the definitive HSCs (Zeng et al., 2019).

By compared to PBMNCs, cHSPCs showed a distinct biology characteristic as chromosome segregation, DNA replication, cell cycle and metabolism (Fig. S8 and Table S4).

To validate our bulk cell RNA-seq data, we performed high-throughput single cell qRT-PCR to detect the critical TFs for HSPCs (Fig. 3H), and the results confirmed that cHSPCs held the definitive HSC signatures. Taken together, these results suggest that cHSPCs exhibited HSPC features.

### Single-cell RNA-Seq analysis elucidates the molecular signatures of cHSPCs

To dissect the transcriptome of cHSPCs at a single-cell resolution, we performed single-cell RNA-Seq analysis using the 10× Chromium platform. Enriched mHSPCs using CD34^+^ microbeads served as positive control. The analysis process was performed as Hao et al., described (2021). Batch analysis showed that the samples from two individual donors exhibited a similar trend of cell clustering, indicating no batch effect (Fig. S9A). An average of 20,752 features were detected, and 50,882 single cells including 45,190 cells in 3DCS were acquired for the downstream analysis after quality control.

**Figure 4:**
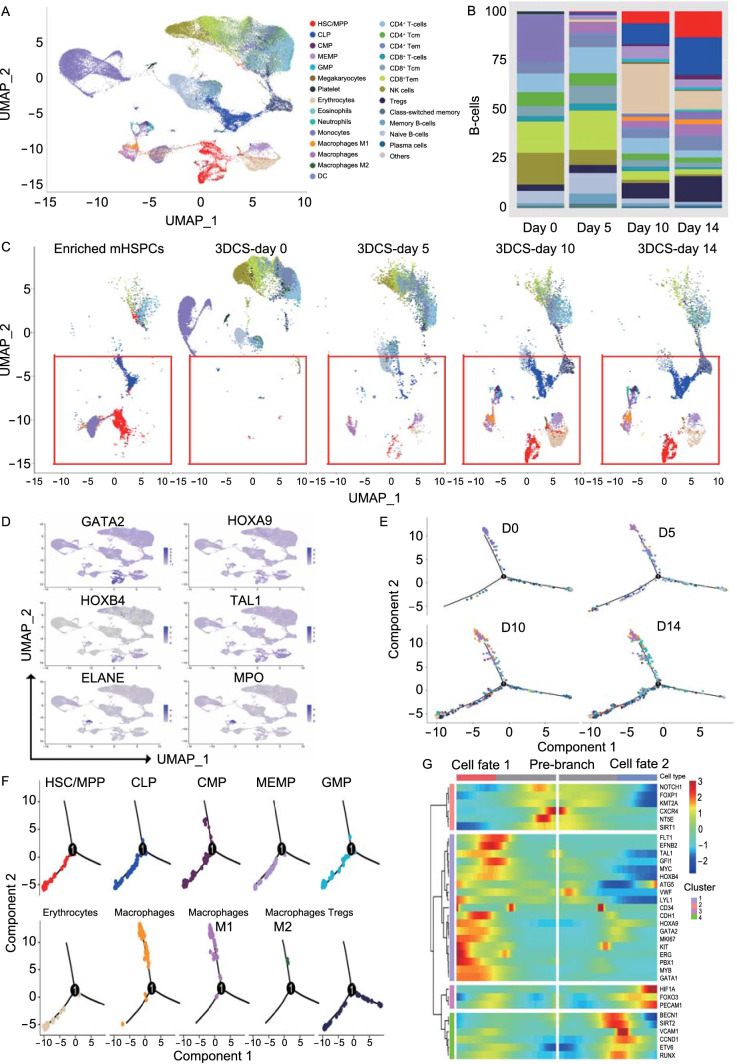
**A single-cell transcriptome atlas for cHSPCs in 3DCS**. (A) The cell types presenting in 3DCS cultures. (B and C) Dynamic changes in hematopoietic subpopulations along the days of culture. The results demonstrated that the gradual increase of HSPCs including HSC, and MPP populations along the culture days (red circle). (D) Analysis of the expression of curated hematopoietic genes in the subpopulations using UMAP visualization. (E) Pseudotime state transition analysis of all cell types along the pseudotime axis defined by Monocle 2. The result demonstrated that mature cells were at early culture days, while HSPCs presented at the late stages. (F) Trajectory development visualization of HSC, MPP, CLP and CMP clusters. (G) Expression pattern of the representative hematopoietic specific TFs including stem and mature genes along the pseudotime axis. Cells (column) are ordered according to the pseudotime development

To determine the single-cell identities, we used SingleR combined with the manual definition according to the expression of specific transcription factors (Aran et al., 2019). 50,882 single cells were annotated to 28 cell types, including HSCs/multipotent progenitors (HSCs/MPPs), common lymphocyte progenitor (CLPs), and common myeloid progenitors (CMPs) (Fig. 4A). The dynamics of each cell population was analyzed along with the culture (Fig. 4B and 4C). At day 0, terminally differentiated cells types were dominant, while HSCs and committed progenitors were very rare. Along with the culture, the terminally differentiated cells gradually decreased, while HSPC subpopulations markedly increased, supporting our hypothesis that 3DCS promoted HSPC expansion. The expression of specific feature genes was visualized through uniform manifold approximation and projection (UMAP) (Figs. 4D and S9B), and heatmap analysis (Fig. S9C).

The developmental trajectories along with hematopoietic development were investigated by Monocle 2 (Trapnell et al., 2014; Qiu et al., 2017a, 2017b) (Figs. 4E, 4F, and S9D). HSCs and committed progenitors were present in the initial state. At the bifurcation point, CLPs and CMPs were separated into two branches, which drove their further differentiation into downstream granulocyte/monocyte progenitors (GMPs), megakaryocyte/erythroid/mast progenitors (MEMPs), macrophages and other cells. Terminally differentiated myeloid cells (monocytes and dendritic cells (DCs)) and lymphoid cells (CD4^+^ T cells and natural killer (NK) cells) were distributed separately at the two bifurcated ends of the tree, which was consistent with the differentiation potential of CLPs and CMPs.

Heatmap with expression of specific genes during HSPC development suggested that TFs related to HSC self-renewal potential (e.g., *NOTCH1*, *TAL1*, *GFI1*, *HOXB4*, *CD34*, *RUNX1*, *GATA2*, and *HOXA9*) were predominantly enriched in the pre-branch populations or in the cells at the junction of pre-branch state to cell fate 1 and fate 2 (Fig. 4G). These data indicate the transition in the cell state from stem to committed progenitors, and finally to differentiated cells, concordant with the conventional hematopoietic differentiation trajectory.

Collectively, these results elucidate that 3DCS promotes expansion of cHSPCs and committed progenitors by upregulation of stemness gene expression.

### 3DCS-derived HSCs/MPPs possess unique biological characteristics

For few cHSCs/MPPs in PB at day 0 (defined as day0-PB-HSCs/MPPs) (11 day0-PB-HSCs/MPPs in total 17,916 singe cells at day0), it made it unreasonable to compare 3DCS-derived HSCs/MPPs (termed as 3DCS- HSCs/MPPs) to day0-PB-HSCs/MPPs. Therefore, we enriched CD34^+^ cells from PBMNCs at day 0 followed by scRNA-seq analysis, and get 81 day0-PB-HSCs/MPPs. We pooled 92 day0-PB-HSCs/MPPs together, and pick up 100 mobilized BM-derived HSCs/MPPs (termed as mHSCs/MPPs), and 3DCS-HSCs/MPPs respectively, for the comparison study.

**Figure 5:**
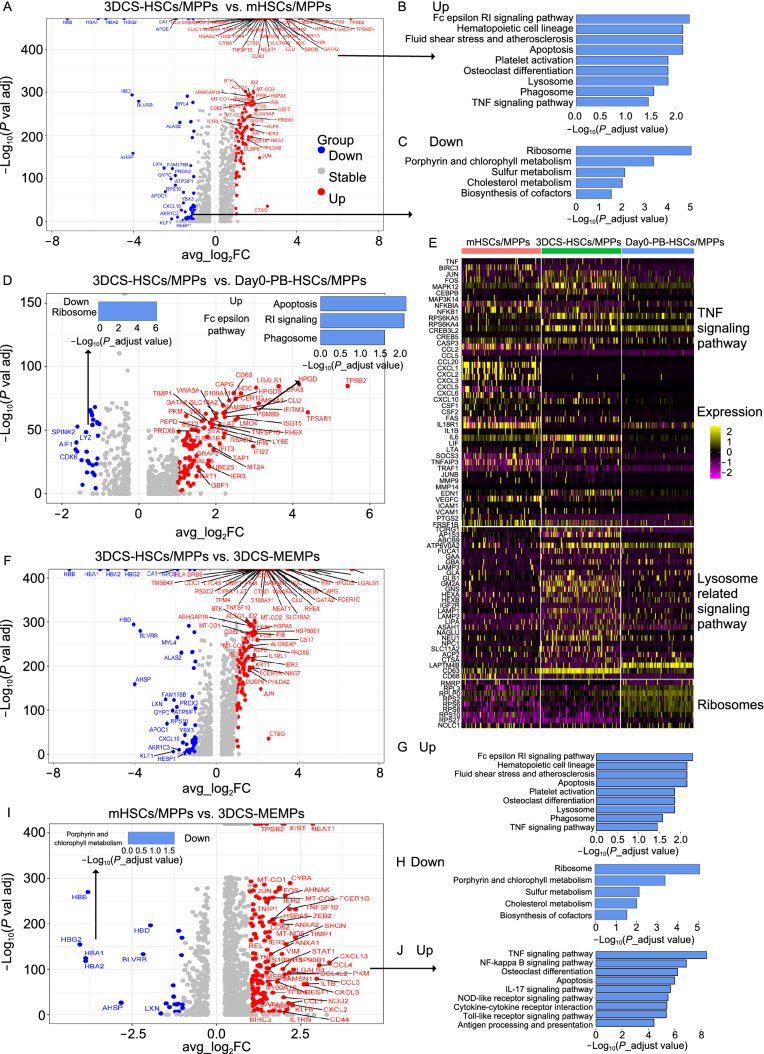
**3DCS-HSCs/MPPs possess unique biological characteristics**. (A) Volcano Plot for DEGs between 3DCS-HSCs/MPPs and mHSCs/MPPs. (B and C) KEGG assay of DEGs between 3DCS-HSCs/MPPs and mHSCs/MPPs. (D) DEG analysis of volcano and KEGG enrichment for 3DCS-HSCs/MPPs versus day0-PB-HSCs/MPPs. (E) Heatmap showed the different expression values of critical molecules for TNF signaling pathway, lysosome-related pathway and ribosomes among 3DCS-HSCs/MPPs, mHSCs/MPPs, and day0-PB-HSCs/MPPs. (F) Volcano assay for DEGs between 3DCS-HSCs/MPPs and day0-PB-HSCs/MPPs. (G and H) KEGG enrichment analysis revealed different regulation pathways in 3DCS-HSCs/MPPs and day0-PB-HSCs/MPPs. (I and J) Volcano and KEGG analysis demonstrated the different gene expression patterns and regulation pathways between mHSCs/MPPs, and day0-PB-HSCs/MPPs. Some genes were labelled with the cutoff of |avg_log2FC| > = 1.5 and *P*_val_adj < 0.05

By comparing 3DCS-HSCs/MPPs with mHSPCs based on DEGs of scRNA-seq, we verified that the pathways as lysosome, osteoclast differentiation, and TNF were enriched in 3DCS-HSCs/MPPs, and pathways for protein synthesis and metabolism process including ribosome and porphyrin and chlorophyll metabolism were down-regulated in 3DCS-HSCs/MPPs, indicating that 3DCS-HSCs/MPPs might be in a state of relatively low metabolism and nutrient stress (Fig. 5A–C). At the same time, 3DCS-HSCs/MPPs also showed some differences with day0-PB-HSCs/MPPs. The pathways as apoptosis, Fc epsilon RI and phagosome were enriched in 3DCS-HSCs/MPPs, however, ribosome pathway was higher in day0-PB-HSCs/MPPs, indicating the different conditions of the inflammatory stress and protein synthesis ability between the groups (Fig. 5D). According to the results, we further analyzed the expression of critical molecules of the pathways as TNF, lysosome, and ribosome in 3DCS-HSCs/MPPs, mHSCs/MPPs and day0-PB-HSCs/MPPs. The results showed that the pathways of TNF and lysosome were up-regulated in 3DCS-HSCs/MPPs, although TNF pathway was also high in mHSCs/MPPs (Fig. 5E). Ribosome pathway was higher in day0-PB-HSCs/MPPs, instructing more active protein synthesis state.

Surprisingly, when we analyzed the differences between 3DCS-HSCs/MPPs and 3DCS-MEMPs (Fig. 5F–H), we found that the most DEGs and the enriched signal pathways were almost the same to those between 3DCS-HSCs/MPPs and mHSCs/MPPs. Additionally, UMAP plots also showed that the adjacent or overlapping spatial mapping of mHSCs/MPPs and 3DCS-MEMPs. Therefore, we further analyzed the differences between mHSCs/MPPs and 3DCS-MEMPs, and found that pathways as TNF, NF-kappa B, and osteoclast differentiation were primarily enriched in mHSCs/MPPs (Fig. 5I and 5J), indicating that the above signal pathways might function in regulating mHSCs/MPPs.

### 3DCS mimics an artificial niche for cHSPC expansion

**Figure 6:**
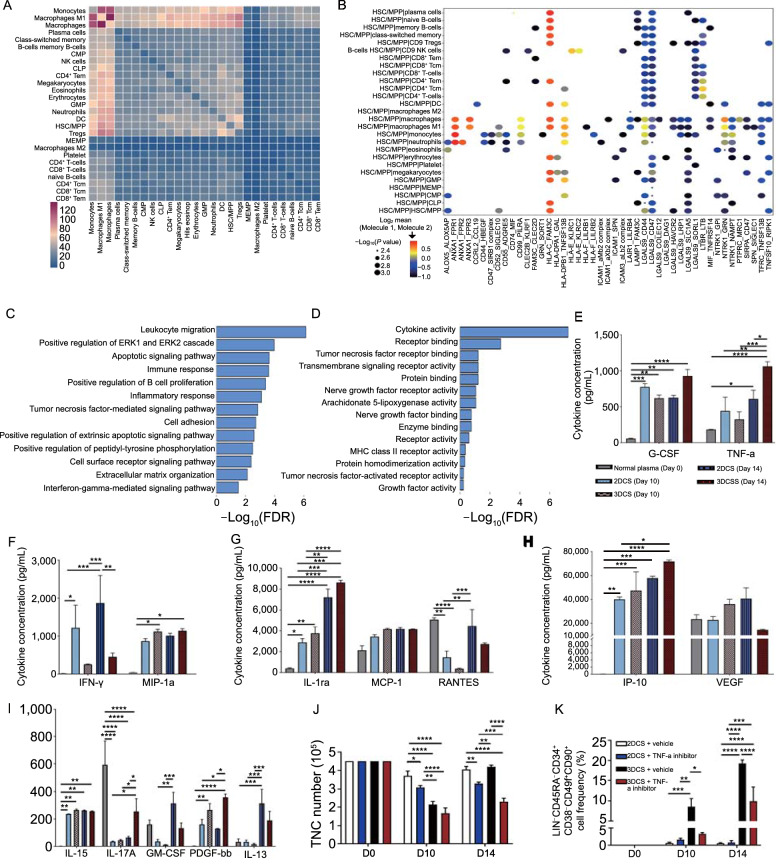
**Cellular interaction and cytokine analysis for 3DCS**. (A) Heatmap demonstrating the total number of ligand-receptor interactions between cell types using CellPhoneDB. (B) Dot plot assays for selected communicating pairs for 3DCS-HSPCs with the other cell types by the cutoff of *P* value < =0.005 and log2 (mean value) > = −1. Circle size represents *P* values. The means of log2 transformation of the average expression level of interacting molecule 1 in 3DCS-HSPCs and interacting molecule 2 in the other cell type are showed with color. (C and D) GO: BP (C) and GO:MF terms (D) enriched in cHSPCs. (E–I) Cytokine array analysis for the samples in 3DCS and in 2DCS. The results showed that the great disparities for cytokine secretion between 3DCS and 2DCS. (J) Apremilast, a TNF-α inhibitor, significantly inhibited TNCs in both 2DCS and 3DCS. (K) The TNF-α inhibitor apremilast significantly inhibited LIN^−^CD45RA^−^CD34^+^CD38^−^CD49f^+^CD90^+^ cHSPCs in 3DCS. Data are the means ± SEM from three replicates. *P < 0.05, **P < 0.01, ***P < 0.001, ****P < 0.0001

To determine the detailed mechanisms underlying cHSPC expansion in 3DCS, we exploited CellPhoneDB to analyze the communicating pairs of PBMNC-derived cHSPCs (Efremova et al., 2020). Among the observed cellular interactions, monocytes, macrophages and macrophages M1 were most active (Fig. 6A). Notably, 3DCS-HSPCs interacted with monocytes, macrophages, and macrophages M1, while they showed less interactions with lymphoid cells. The HLA-C_FAM3C, TFRC_TNFSF13B, and MIF_TNFRSF14 pairs were abundantly observed among the interactions among 3DCS-HSPCs with the niche (Fig. 6B). The results showed that the interactions of 3DCS-HSPCs were distinct from those observed in models of interactions among HSCs and niche stromal cells during embryonic hematopoiesis (Zeng et al., 2019). GO biological process (BP) enrichment and molecular function (MF) analyses revealed that function enriched in cHSPCs was related to responses to multiple stimuli, cell adhesion, and cell proliferation (Fig. 6C and 6D). In particular, the tumor necrosis factor (TNF) activities were predominantly up-regulated, suggesting that these pathways might function in 3DCS-HSPCs.

To validate our hypothesis, we performed cytokine array analysis (Figs. 6E–I, S10A a and b). The results showed that cytokines as PDGF-bb, IL-12 and IL-17a were specifically enriched in 3DCS, whereas cytokines such as IFN-γ, IL-13 and RANTES were higher in 2DCS. Of note, the level of TNF-α in 3DCS was significantly higher than that in 2DCS, consistent with a previous study that TNF-α is a major pro-survival and pro-regeneration factor for HSC emergence and fate determination (Yamashita and Passegue, 2019). UMAP analysis showed that TNF was secreted by the cells as macrophages, and monocytes (Fig. S10B). To further test the effect of TNF on 3DCS-HSPCs expansion, we applied TNF-α inhibitor apremilast into 3DCS and 2DCS. The results showed that apremilast significantly reduced the number of the total nucleated cells (TNCs) and the frequency of LIN^−^CD45RA^−^CD34^+^CD38^low/−^CD49f^+^CD90^+^ cHSPCs in 3DCS (Fig. 6J and 6K), indicating that TNF-α regulated 3DCS-HSPCs survival and expansion.

In summary, these results demonstrate that 3DCS might function as an artificial niche to provide signals such as TNF, which regulates the expansion or survival of 3DCS-HSPCs.

### 3DCS efficiently expands cHSPCs in peripheral blood from patients who fail in BM-HSPC mobilization

HSC transplantation (HSCT) presents significant clinical advantages (Mohty et al., 2014). Granulocyte colony-stimulating factor (G-CSF) or CXCR4 antagonist are administered into donors to mobilize their BM-HSCs into the peripheral blood (Petit et al., 2002). However, 5%–46% of donors or patients fail in HSC mobilization (Perseghin et al., 2009). Hence, we next tested whether our 3DCS could expand the rare cHSPCs for those patients.

**Figure 7:**
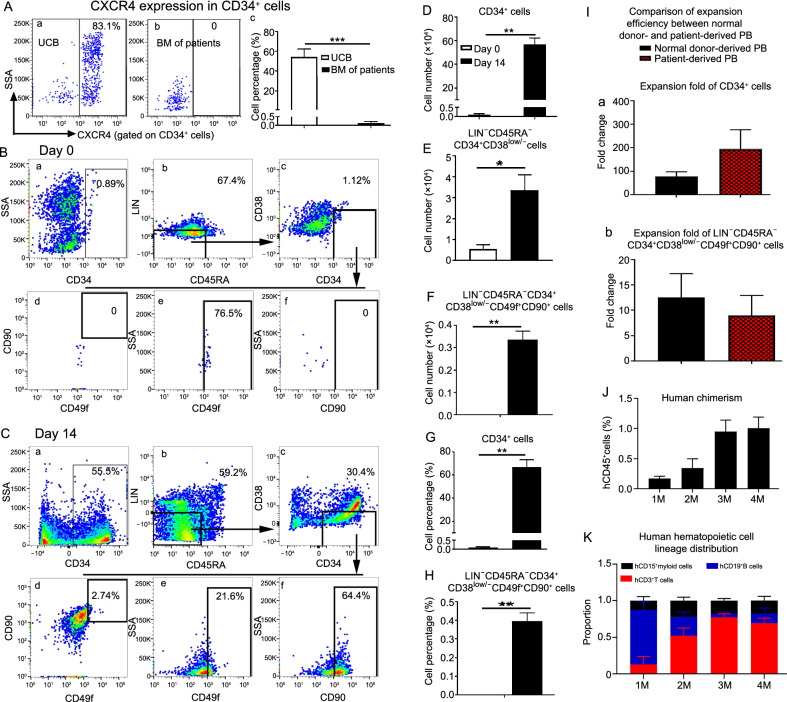
**3DCS efficiently amplified rare cHSPCs in PBMNCs derived from patients who failed to show HSPC mobilization from the BM**. (A) Expression level analysis of CXCR4 in CD34^+^ cells in the BM of the patients. The results showed that the CXCR4 expression level was very low compared to that in CD34^+^ cells in UCB, verifying the possible mechanism of mobilization failure. (B and C) Representative flow cytometry plots showing cHSPC subpopulations in patient-derived PBMNCs on day 0 (B) and on day 14 (C). The results demonstrated that 3DCS efficiently amplified rare cHSPCs in the PBMNCs of patients who failed to show HSC mobilization. (D–H) The kinetics of the numbers and percentages of the subpopulations of cHSPCs during the days of culture. (I) Comparison of amplification efficiency between volunteer- and patient-derived PB. (J) Kinetics of human chimaerism in the transplanted mice. (K) The kinetic distribution of human hematopoietic cell lineages *in vivo*. UCB, umbilical cord blood. Data are the means ± SEM from 3–6 replicates. *P < 0.05, **P < 0.01, ***P < 0.001, ****P < 0.0001

We collected BM and PB from three patients who previously failed in HSC mobilization (Table S5). We first detected the expression of CXCR4 in CD34^+^ cells, and found that the expression level of CXCR4 was much lower than that in the umbilical blood group (Fig. 7A). Then, we cultured 1.2 × 10^6^ patient-derived PBMNCs in 3DCS. After 14 days, we analyzed the cell number and the percentage of cHSPCs (Fig. 7B and 7C). For the patient 1, we found that the percentage of CD34^+^ cells increased from 0.32% to 66.7%, and the absolute number increased from 0.384 × 10^4^ to 56.7 × 10^4^, representing 208-, and ~148-fold increase respectively. Similar results were also observed from the other two patients. CD34^+^, and LIN^−^CD45RA^−^CD34^+^CD38^low/−^CD49f^+^CD90^+^ cHSPC subsets were also significantly expanded (Fig. 7D–H). Kinetics of cHSPCs were similar between the normal donor- and patient-derived cHSPCs (Fig. 7I a and b). Importantly, the patient-derived cHSPCs held repopulation capacity in immunocompromised mice (Fig. 7J). The distribution of human hematopoietic lineages was calculated (Fig. 7K). Collectively, these results suggest that 3DCS could expand rare cHSPCs for patients with HSC mobilization failure.

## DISCUSSION

The expansion of functional HSPCs for clinical transplantation is a long-sought goal in regenerative medicine. Several groups have recently applied three-dimensional materials to prompt *ex vivo* expansion of CB- and BM-derived HSPCs or to induce pluripotent stem cell commitment to HSPC-like cells (Xu et al., 2016; Bai et al., 2019; Wilkinson et al., 2019a; Shan et al., 2020, 2021). In the study, we presented that 3D-niche using polypeptide-forming three-structure had great advantage in capturing and expanding rare cHSPCs in steady-state peripheral blood with long term repopulation capacity. Because PBMNCs are collected in a simple and noninvasive manner compared to mHSPCs or to BM-HSPCs, 3DCS might benefit for the clinic treatment, especially for those patients who fail in HSPC mobilization.

Although sharing similar morphology and functionality, cHSPCs and mHSPCs still exhibited functional differences in the *in vivo* multilineage and repopulation potential. In the primary transplantation, cHSPCs produced more T cells but fewer B cells in recipients than mHSPCs did. Following secondary transplantation, cHSPCs were prone to T lymphocyte-biased repopulation, whereas mHSPCs preferentially showed myeloid differentiation.

3DCS regulated the proliferation of cHSPCs and the maintenance of their long-term repopulating capacity by promoting the expressions of stemness-associated genes. Bulk RNA-Seq and high-throughput qRT-PCR analyses showed that TFs, such as *GFI1B*, *HOXA5*, *HOXA7*, and *TAL1*, which are essential for HSC self-renewal, were expressed at high levels in 3DCS, which endowed cHSPCs with high reconstitution capacity. *AhR*, which protein antagonist SR1 has been shown to successfully expand human HSCs (Singh et al., 2009) (Boitano et al., 2010), was much lower in 3DCS than in 2DCS, indicating the potential molecular mechanism involved in 3DCS-derived HSPCs. *EPX*, responsible for immune cell hypersensitivity reactions, was expressed at a higher level in cHSPCs than in mHSPCs, indicating that it might function as a mediator of the hypersensitivity reaction of HSPCs circulating in PB or under the culture pressure *in vitro* (Papayannopoulos et al., 2010). Furthermore, bulk RNA-Seq and qRT-PCR analysis demonstrated that cHSPCs expressed a higher level of hemogenic endothelial cell and mesoderm markers such as *CD34*, *KDR*, *TEK*, *CDH5*, *CDH1*, *HEY2*, *APLENR*, and *SOX17*, implying that 3DCS-derived cHSPCs might share the molecular signature with the hemogenic endothelial cells. The higher expression of *KI67*, *c-MYB*, *c-KIT*, and *CYCLIND1* indicated stronger proliferation activity in cHSPCs in 3DCS than those in 2DCS and PBMNCs. The expression of *TEK*, *CDH1*, *CDH5* and *CXCR4* in 3DCS-derived CD34^+^ cells correlated with the homing and interaction capacities between 3DCS-derived cHSPCs and their niche. Although we found that the cHSPCs in 3DCS exhibited higher expression of genes related to the cell cycle, translation and mitochondrial metabolism, these cells could engraft and give rise to all three lineages after rigorous serial transplantation.

Our polypeptide-forming three-dimensional system provided multiple niche signals which mediated the interaction between cHSPCs and the other cells. Single-cell RNA-Seq, cytokine arrays, and small molecule inhibition analyses revealed that TNF-α was responsible for cHSPC expansion in 3DCS. The impact of TNF-a on HSC self-renewal or stemness potential remains controversial, since both promoting and inhibitory effects are observed, depending on the experimental settings (Pearl-Yafe et al., 2010; Pronk et al., 2011; Ishida et al., 2017; Yamashita and Passegue, 2019). However, emerging studies recently have shown that TNF-a is an essential regulator of HSC ontogeny during embryonic development (Espin-Palazon et al., 2014) and supports murine hematopoietic progenitor function in the early stages of engraftment (Pearl-Yafe et al., 2010). Moreover, TNF-a promotes HSC survival and myeloid differentiation by activating NF-kB-dependent gene program that primarily prevents necroptosis, induces immunomodulatory functions, and poises HSCs for myeloid cell production, which are critical for HSC response to inflammatory stress (Yamashita and Passegue, 2019). In our study, we found the cells as macrophages, Tregs and B cells in 3DCS secreted TNF. The inhibition of TNF-α suppressed the colony forming ability of cHSPCs, and decreased the percentage and number of LIN^−^CD45RA^−^CD34^+^CD38^low/−^CD49f^+^CD90^+^ HSPC populations. However, it remains to be determined whether TNF-α concentration is dependent on the culture milieu such as polypeptide-forming 3D structure, or/and the factor cocktails applied in the study, or/and the stromal cells produced in 3DCS. Additionally, it remains to be explored whether the repopulation capacity of 3DCS-expanded cells is compromised and whether TNF-α-driven mechanisms are correlated with HSC stemness-related genes under TNF-α suppression condition, to confirm that TNF-α is the key factor involved in the *in vitro* expansion and function maintaining of cHSPCs.

Our new findings have shown that 3DCS facilitates the expansion of cHSPCs with long-term repopulation abilities, which provides a promising strategy for the clinical application of HSC therapy, drug screens, disease modeling, and gene editing, particularly for the patients/donors who are at high risk for failure in HSC mobilization.

## CONCLUSIONS

In this manuscript, we have applied the self-assembly polypeptide-forming three-dimensional culture system (3DCS) to successfully expand the rare circulating HSPCs with repopulation capabilities. We found that the frequency and cell number of CD34^+^ cells increased by 125- and 70-fold respectively after 14 days culture in 3DCS compared to day 0. Comprehensive *in vitro* colony forming unit assays and *in vivo* serial transplantation were performed to prove the 3DCS-derived cHSPCs exhibited self-renewal and multi-lineage differentiation capacities. Further, we combined bulk/single cell RNA-Seq, high-throughput PCR, cytokine array and other assays, and elucidated that the 3DCS fabricated an immunomodulatory microenvironment, which secreted cytokines such as TNF to support cHSPC survival. 3DCS-derived HSPCs possessed unique biological characteristics with higher level of pathways as TNF and lysosome compared to BM-derived HSPCs and PB-derived HSPCs. Finally, we validated that 3DCS could also promote the expansion of cHSPCs in patients who previously failed in HSC mobilization. Taken together, our study provides a new cell source for HSPC transplantation, drug screens, disease modeling, and gene editing.

## METHODS

### Preparation of normal PBMNCs

The protocol was approved by the Ethical Committee of Zhejiang University, and written informed consent was obtained from all volunteers. Approximately 10–15 mL of healthy blood was extracted using single-use containers for human venous blood specimen collection (Becton, Dickinson and Company) with anticoagulation properties. Peripheral blood mononuclear cells (PBMNCs) were isolated via Ficoll-Hypaque (Sigma) gradient centrifugation at 400 ×*g* for 25 min. The cell pellets were washed with phosphate-buffered saline (PBS) free of Ca^2+^ and Mg^2+^ two times to remove remnants of Ficoll-Hypaque because of its toxicity and then prepared for 3DCS.

### Establishment of 3DCS

The biomimetic cell culture material used in the study was purchased from Corning® PuraMatrix™ Peptide Hydrogel (Life Sciences, USA). The operation was processed as previous description (Xu et al., 2016). Briefly, the cells were prepared and mixed with the diluted hydrogel. Medium consisted of commercial base culture medium (StemSpan™ SFEM II, STEMCELL, Canada) plus sets of factors including 100 ng/mL SCF, 100 ng/mL FLT3L, 20 ng/mL TPO, 2 ng/mL VEGF, 20 ng/mL IL-3, 20 ng/mL IL-6, 1 µmol/L SR1, and 25 µg/mL Vc. Cultures of PBMNCs in a two-dimensional culture system (2DCS) served as controls, which contained the same culture media as 3DCS except for the addition of the polypeptide material.

### Optimization of 3DCS

At the beginning of the experiment, we optimized the conditions for 3DCS. The strategies were developed from the basic culture medium for HSPC expansion with SFEM II supplementation with five hematopoietic growth factors (SCF, FLT3L, TPO, IL3 and IL6), referred to as the 5 factors. To this basic medium, we added VEGF, SR1, Vc, VEGF + SR1, VEGF + Vc, SR1+ Vc, VEGF + SR1+ Vc separately. The cell numbers and the proportion of HSPCs were evaluated as indicators of the efficiency of the systems described above, and the best combination of conditions was selected for the next experiment in the study.

### Serial transplantation in immunocompromised mice

The animal protocol was approved by the Institutional Animal Care and Use Committee of Zhejiang University. PBMNCs were cultured for 14 days in 3DCS, and were treated with 0.25% trypsin with 0.02% EDTA, followed by washing with PBS. After filtration through 40 µm strainers, about 7 × 10^5^ cells were administered to 7~8-week-old NOD-Prkdc^em26Cd52^Il2rg^em26Cd22^/Nju (NCG) mice irradiated with 1.8 Gray. The chimerism of human cells was monitored at the indicated time. Sixteen weeks after transplantation, the recipients were sacrificed, and blood tissues were processed for flow cytometry assays. The samples in 2DCS and mHSPCs (mobilized BM-derived HSPCs) served as control groups.

For the second transplantation assay, a quarter of the BM was isolated from the recipients and infused into NCGs. After 16 weeks, the PB, BM, liver and spleen were harvested and processed to detect human cell chimerism and the percentage thereof.

### Single-cell RNA sequencing analysis

Single-cell RNA sequencing (scRNA-seq) analysis was applied to detect the dynamic changes in cell types in 3DCS with PBMNCs derived from two donors. Samples were collected on day 0, day 5, day 10 and day 14 and prepared for subsequent droplet-based scRNA-seq production according to the manufacturer’s protocol (10× Genomics, CA, USA). For single-cell RNA sequencing, libraries were prepared using Chromium Single Cell 3′ Reagent Kits v.2. Expression profiling datasets; these libraries have been deposited at Gene Expression Omnibus (GEO, www.ncbi.nlm.nih.gov) and are available under accession number GSE153421.

### Cytokine array analysis

The Bio-Plex Pro Human Cytokine Grp I Panel including IL-1b, IL-2, IFN-g, TNF-a, and G-CSF was applied to detect cytokine secretion with the Bio-Plex MAGPIX System (Bio-Rad) according to the manufacturer’s instructions. Measurements were performed using the Bio-Plex MAGPIX Multiplex Reader. Plasma samples were collected and served as data for day 0. Supernatant fractions were collected at the indicated times for the measurement of cytokine secretion.

### Statistical analysis

The data are presented as the means and standard deviations (SDs) or standard errors of the mean (SEM). GraphPad Prism 7 was applied for all statistical analyses. Two-way ANOVA followed by Tukey’s post hoc test was used for comparisons among multiple groups, and Student’s t test was used for comparisons between two groups. Values of *P* < 0.05 were considered to indicate statistically significant differences.

## Supplementary Information

Below is the link to the electronic supplementary material.

Supplementary file1 (XLSX 10 KB)

Supplementary file2 (XLS 86 KB)

Supplementary file3 (XLS 49 KB)

Supplementary file4 (XLS 1046 KB)

Supplementary file5 (XLS 20 KB)

Supplementary file6 (XLS 11 KB)

Supplementary file7 (PDF 31621 KB)
