# Supplementary material for: Efficient expansion of rare human circulating hematopoietic stem/progenitor cells in steady-state blood using a polypeptide-forming 3D culture
Source: Protein Cell. 2022 Mar 1;13(11):808–24. doi: 10.1007/s13238-021-00900-4 (PMC9237197; doi:10.1007/s13238-021-00900-4)
Supplement: Supplementary file 7 — Supplementary file7 (PDF 31621 KB) [file 13238_2021_900_MOESM7_ESM.pdf]

Supplemental information for

Efficient expansion of rare circulating human hematopoietic stem/progenitor cells in peripheral blood using an artificial 3D system

Methods

Cell growth and apoptosis analysis. Ki67 detection was performed to estimate cell proliferation. After fixation with 70% precooled ethanol, the samples were stained with Alexa Fluor488 anti-human Ki67 (BioLegend) followed by washing in PBS and FCM detection. A Countstar®Cell Analysis System (ALIT Life Science) was applied for cell number counting according to the manufacturer's instructions. Cell apoptosis and death analysis was performed with an Annexin V/7-ADD Apoptosis Detection Kit (PEPROTECH, USA). The targeted samples were prepared according to the manufacturers' instructions. After washing with PBS, the cells were analyzed via FCM. The staining pattern was evaluated to determine cell viability, apoptosis, or death.

The cells cultured in 2DCS served as control groups and were processed using the same procedures. All the above assays were performed 3-4 times.

Flow cytometry analysis. The phenotypes were detected with the following human antibodies purchased from BioLegend: CD34-PE-Cy7, CD38-PerCP-Cy5.5, CD90-BV510, LIN-FITC (CD2, CD3, CD4, CD7, CD8, CD10, CD11b, CD14, CD19, CD20, CD56, and CD235a), CD45RA-APC, and Ki67-AF488. The CD49f-PE antibody was purchased from BD. The samples were collected from 3DCS by pipetting up and down to destroy the structure. The cells were collected and digested with trypsin-EDTA solution (0.25% trypsin and 0.02% EDTA) and then washed with PBS containing 2% fetal bovine serum (FBS) 2-3 times. Clumps were removed with 40 μm cell strainers. A total of 5×10^5 single cell suspensions were incubated with

the specified antibodies for 30 min at room temperature, followed by washing with PBS 2-3 times, and were then prepared for FCM analysis (BD Fortessa). The control groups were subjected to the same procedure. In the transplantation assay, the other antibodies used for human chimerism detection were CD45-APC-H7, CD19-APC, CD3-PerCP-Cy5.5, CD15-BV605, CD34-PE-CY7, CD4-FITC, CD8-BV510, and an antibody for mouse CD45-PE-CF594, which was applied in the tests to define the repopulation ability of cHSPCs, including the second transplantation assay. For the detection of the samples from the patients, in addition to the above antibodies, CXCR4-PE was ordered from BioLegend. FlowJo 7.6 Software was applied for statistical analysis.

Scanning electron microscopy (SEM) and transmission electron microscopy (TEM) analysis. Scanning electron microscopy (SEM) analysis was performed to observe the morphology of the cells in detail. The protocol was carried out according to the instructions. Briefly, the samples were collected at the indicated time points and fixed with a 2.5% glutaric dialdehyde solution overnight. After washing with PBS, the cells were treated with 1% osmic acid for 1.5 h and then dehydrated in a series of 30%, 50%, 70%, 80%, 90%, 95%, 100%, and 100% ethanol, for 20 min in each solution. After drying and gold coating, the samples were observed with a Nova NanoSEM 450 SEM (FEI).

Transmission electron microscopy (TEM) analysis was carried out to visualize the ultrastructure of the cells. The samples were fixed with a 2.5% glutaric dialdehyde solution for more than 4 h. After washing with PBS, the cells were treated with 1% osmic acid for 1 h and then washed with distilled water 2-3 times. After fixation in 2% uranium acetate, the cells were dehydrated in a series of 50%, 70%, 90%, and 100% ethanol for 10-15 min in each solution and were then incubated in 100% acetone 2 times for 10-15 min each. After permeation, entrapment,

polymerization, and uranyl acetate-lead citrate staining, the samples were observed with Cryo TEM (Tecnai Spirit).

CD34⁺ HSPC enrichment and colony forming unit (CFU) assay. The protocol was approved by the Ethical Committee of Zhejiang University. All volunteers were informed and provided written consent. An EasySep CD34 positive selection kit (StemCell Technologies, Canada) was applied for $CD34^+$ cell isolation with an EasySep® Magnet device (StemCell Technologies, Canada) according to the manufacturer's instructions.

A CFU assay was carried out to detect the multilineage ability of the samples in 3DCS. Approximately 1000 $CD34^+$ cells were seeded in low-adherence dishes in Methocult 4434 (StemCell Technologies, Canada). The cells were incubated at 37 ℃ in a humidified atmosphere with 5% CO_2 for approximately 2 weeks. The cultures were performed in triplicate. Colonies belonging to burst-forming unit-erythroid (BFU-E), colony-forming unit-granulocyte/macrophage (CFU-GM), colony-forming unit-macrophage (CFU-M) and colony forming unit-granulocyte/erythroid/macrophage/megakaryocyte (CFU-GEMM) lineages were observed and scored according to their cellular morphology and the extent of visible cell content under an inverted microscope (Olympus). Cells isolated from 2DCS and mHSPCs served as control groups.

Bulk-cell RNA-Seq. To determine the cellular identity within 3DCS, we performed a gene expression profiling assay based on the bulk transcriptome. Total RNA was extracted from the cells, followed by mRNA enrichment with poly-T oligo-attached magnetic beads. Cells from 2DCS and PBMNCs served as controls (the number of repetitions for each group, n = 3). mHSPCs were purified as a positive control (n = 4).

Raw sequencing data were generated using the Illumina HiSeq™ platform, and 125 bp/150 bp

paired-end reads were selected. The expression profiling datasets have been deposited in the Gene Expression Omnibus (GEO, www.ncbi.nlm.nih.gov) and are available under the accession number GSE122682.

Differentially expressed genes (DEGs) and enrichment analysis. Clean reads were obtained by removing reads containing adapter and poly-N sequences, and low-quality reads from the raw data. HTSeq (version 0.6.0) was applied to calculate the reads mapped to each gene. Based on the length of the genes and read counts, FPKM (fragments per kilobase of transcript sequence per million base pairs sequenced) values were calculated.

Based on the FPKM values, DEGs were analyzed with the DESeq2 R package (1.10.1). *P*-values were adjusted using the Benjamini and Hochberg approach to control the false discovery rate (FDR). Genes with an adjusted *P*-value < 0.05 identified by DESeq2 were assigned as differentially expressed.

Principal component analysis (PCA) and the hierarchical clustering method (Euclidian distance, average linkage) were applied to explore the expression patterns among the groups. Gene Ontology (GO) and Kyoto Encyclopedia of Genes and Genomes (KEGG) pathway enrichment analysis were performed with the clusterProfiler R package or DAVID bioinformatics resources (http://www.david.ncifcrf.org/). GO terms and KEGG pathways with adjusted *P*-values < 0.05 were considered significantly enriched.

According to the log_2FPKM transformation values, we demonstrated the contribution of the specific transcription factors (TFs) regulating hematopoiesis development through the modulation of genes such as heptad genes and endothelial-to-hematopoietic transition (EHT)-, HSPC-, and mature hematopoietic cell-related genes. The epitope expression pattern was also explored with selected surface markers showing high contributions to hematopoiesis

development.

Gene set enrichment analysis (GSEA). GSEA was performed to determine the priori defined sets of gene enrichment with hypergeometric testing between the separating groups. To identify the gene sets of interest with custom annotations, we targeted KEGG pathways (http://www.genome.jp/kegg/pathway.html) or the Molecular Signatures Database (MSigDB) in GSEA (http://software.broadinstitute.org/gsea/msigdb/search.jsp). The genes were labeled showing significant differences at an FDR < 0.05 and adjusted *P* value < 0.05.

High-throughput quantitative real-time polymerase chain reaction (qRT-PCR) reaction. To verify the reproducibility of the targeted results for the cell RNA-Seq study, we performed high-throughput qRT-PCR detection. A set of 46 genes that play dominant roles during hematopoiesis development were selected and evaluated. The primers are shown in Supplemental table 6. The procedure was carried out as described in our previous report (*25*). The cDNA products of each cell were sorted according to the Ct values of β-actin, and more than 20 samples from each cell were selected for the analysis of the kinetic expression of the target genes with the AceQ qPCR SYBR Green Master Mix kit (Vazyme, Nanjing, China). Transcription abundance was presented as $\log_2\Delta(35–Ct)$ transformation values for heatmap analysis.

Single-cell RNA-seq data processing. Sequencing data from 10× Genomics were processed with CellRanger software (version 3.1) with default mapping arguments. The Seurat package (version 3.1.0) in R (version 3.5.3) was applied to process the single-cell datasets. Low-quality cells were filtered with the parameters min. cells = 3 and nFeature_RNA > 200 & nFeature_RNA < 4000 & nCount_RNA > 200 & percent.mt < 20. The integrated analysis of two donor-derived datasets was carried out to remove batch and individual differences using SCTransform. A logarithmic transformation was applied to the UMI count matrix with a scale

factor of 10,000. The method for the identification of highly variable features was vst, and nfeatures = 2000. PCA was performed using the nfeatures identified by the FindVariableFeatures function, and significant PCs were selected using the elbow method to perform dimension reduction and clustering. UMAP was used for dimension reduction and the visualization of cell clusters, which was calculated with the function Find Clusters using the default parameters except for “resolution” = 1.8.

Construction of single-cell trajectories with Monocle 2. The pseudotime trajectory was constructed with the Monocle 2 package (v 2.4.0). PCAs were chosen for gene ordering, and the discriminative dimensionality reduction with trees (DDRTree) was applied for dimension reduction. Significant DEGs were identified to investigate the different patterns of gene expression along pseudotime trajectories.

Supplemental Figure legends

Supplemental Figure 1. Optimization assay of 3DCS demonstrating the most efficiency of the combination of VEGF, SR1, vitamin C plus 5 factors for cHSPC expansion. Different combinations of growth factors and cytokines were tested for 3DCS optimization. The results showed that the combination of three small molecules plus 5 factors significantly increased both the percentages and absolute numbers of HSPC subpopulations. Data are the means ± SD from four replicates. ****$P < 0.0001$, ***$P < 0.001$, **$P < 0.01$, *$P < 0.05$.

Supplemental Figure 2. Various morphology presentation, Ki67 expression assay. (A) Distinctly various colonies were formed in 3DCS. **(B)** Cellular growth analysis. **(C-D)** Kinetic analysis for cell percentage of cHSPC subsets. **(E)** Fold change comparison for cell percentages of cHSPC subpopulations in 2DCS and 3DCS. Data are the means ± SD from four replicates. ****$P < 0.0001$, ***$P < 0.001$, *$P < 0.05$.

Supplemental Figure 3. Representative flow cytometry analysis of repopulating assay. (A-D) Representative flow cytometry analysis for repopulation potential of 3DCS-derived cells. **(E-H)** Representative flow cytometry analysis for repopulation potential of mHSPCs in NCGs.

Supplemental Figure 4. Second transplantation confirmed long-term repopulating potential for 3DCS-derived cells. Representative flow cytometry analysis for long term repopulation potential of 3DCS-derived cHSPCs **(A-D)**, and positive control mHSPCs **(E-H)** for the second transplantation in NCGs.

Supplemental Figure 5. Gene set enrichment analysis by comparison 3DCS with 2DCS. (A) Venn diagram showing the number of DEGs among the groups. **(B-C)** Enrichment analysis of GO terms for the molecular function of DEGs between 3DCS and 2DCS. **(D)** GSEA enrichment analysis for 3DCS to 2DCS.

Supplemental Figure 6. Gene profiling analysis showed the similarity of 3DCS-derived cHSPCs with mHSPCs. (A) Volcano analysis for DEGs between 3DCS and 2DCS. Some genes were labelled with the cutoff of |log2foldchange| > 5 and *p-adj* < 0.05. **(B-C)** KEGG enrichment analysis showed that 3DCS-derived cells had more metabolic activity than mHSPCs did. **(D)** GSEA analysis for 3DCS and mHSPCs showed cHSPCs held more metabolic activity than mHSPCs did.

Supplemental Figure 7. cHSPCs held the similar expression profile of HSPC-specific surface markers to mHSPCs, while there was a great disparity between 2DCS-derived cells and mHSPCs.

Supplemental Figure 8. cHSPCs in 3DCS held stem features compared with initial PBMNCs. **(A)** Volcano plot assay for DEGs between the samples in 3DCS and initial PBMNCs. Some genes were labelled with the cutoff of |log2foldchange| > 5 and *p-adj* < 0.05. **(B-C)** GO

term analysis showed down-regulated (B), and up-regulated (C) enrichment in the cells in 3DCS compared to PBMNCs. **(D-E)** GSEA demonstrated positive snapshots (D a-h), and negative snapshots (E a-h) of enrichment in 3DCS-derived cHSPCs.

Supplemental Figure 9. Single-cell analysis for the clusters in 3DCS. (A) Batch analysis for the 2 donor samples demonstrated the same kinetics, indicating the stability of 3DCS in the culture of PBMNCs. **(B)** Curated gene expression for identifying cell types using UMAP visualization. **(C)** Heatmap visualizing expression of the specific genes related to the defined sub-populations in 3DCS. **(D)** Trajectory analysis for the sub-populations in 3DCS.

Supplemental Figure 10. 3DCS produced a special hematopoietic niche supporting cHSPC survival and expansion *in vitro*. (A) Analysis of cytokine secretion revealed an immunosuppressive niche for 3DCS in prompting cHSPC survival and expansion. **(B)** Dynamic change analysis of certain cell types secreting TNF-α. The results demonstrated that a variety of cell types as macrophages, Tregs, and memory B-cells/class-switched memory B cells produced TNF-α in 3DCS.

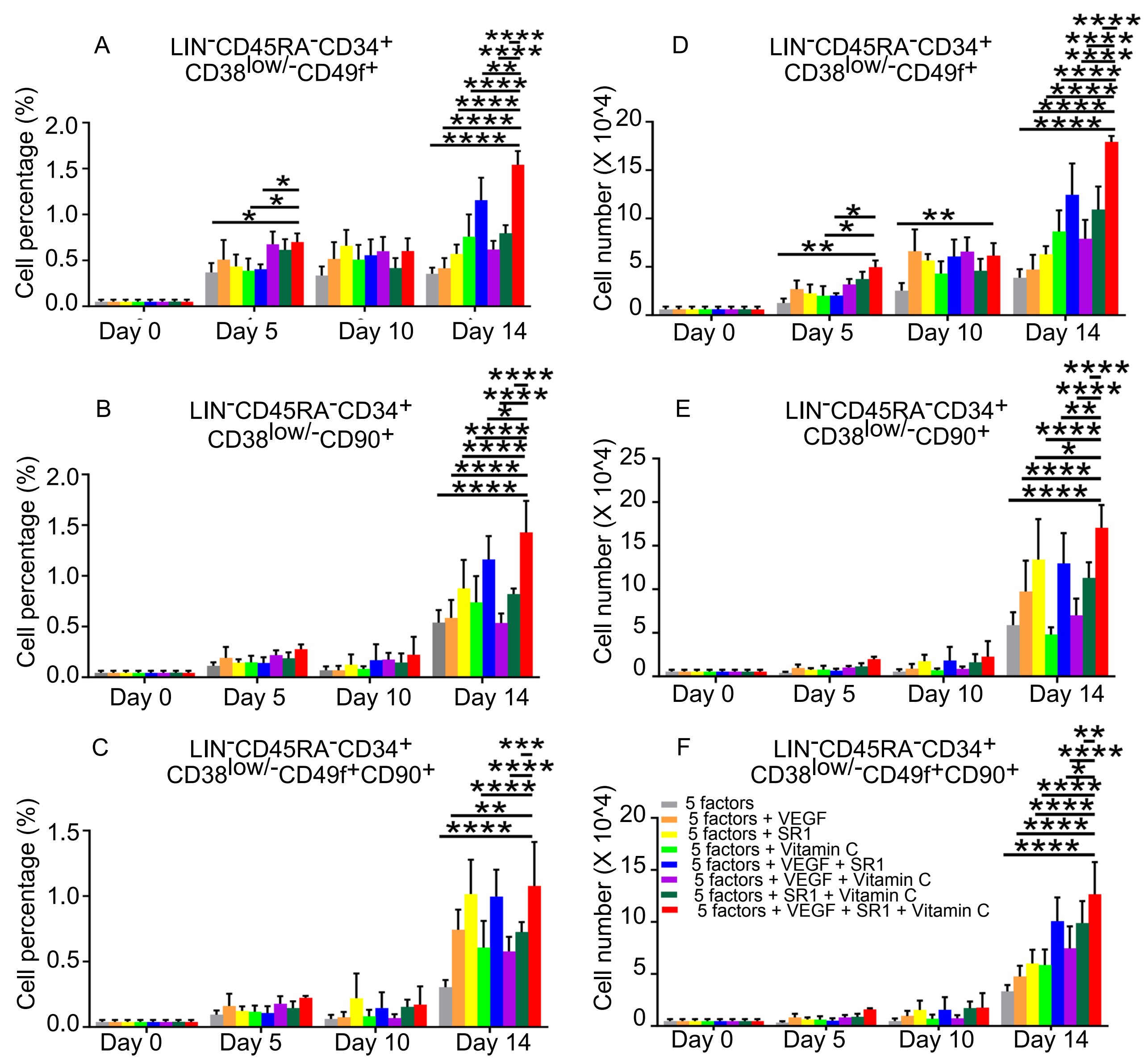

A
LIN⁻CD45RA⁻CD34⁺ CD38low/-CD49f⁺
B
LIN⁻CD45RA⁻CD34⁺ CD38low/-CD90⁺
C
LIN⁻CD45RA⁻CD34⁺ CD38low/-CD49f⁺CD90⁺
D
LIN⁻CD45RA⁻CD34⁺ CD38low/-CD49f⁺
E
LIN⁻CD45RA⁻CD34⁺ CD38low/-CD90⁺
F
LIN⁻CD45RA⁻CD34⁺ CD38low/-CD49f⁺CD90⁺
Cell percentage (%)
Cell number (X 10^4)
Day 0
Day 5
Day 10
Day 14
5 factors
5 factors + VEGF
5 factors + SR1
5 factors + Vitamin C
5 factors + VEGF + SR1
5 factors + VEGF + Vitamin C
5 factors + SR1 + Vitamin C
5 factors + VEGF + SR1 + Vitamin C

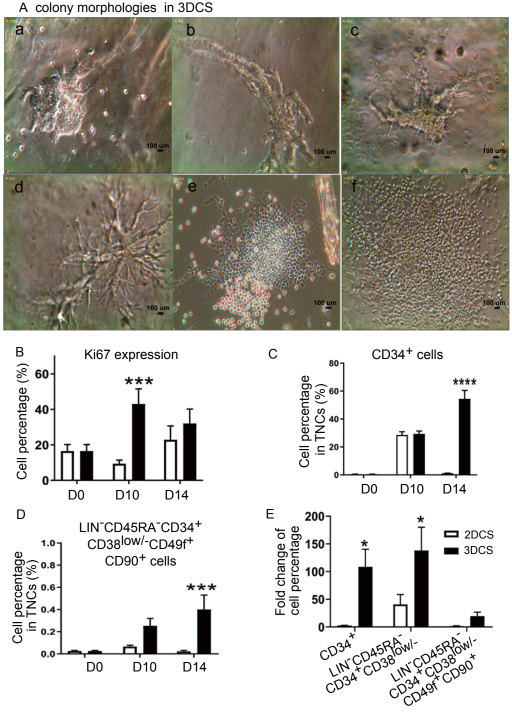

A colony morphologies in 3DCS
a
b
c
d
e
f
100 um
B
Ki67 expression
Cell percentage (%)
D0
D10
D14
C
CD34+ cells
Cell percentage in TNCs (%)
D
LIN-CD45RA-CD34+ CD38low/-CD49f+ CD90+ cells
E
Fold change of cell percentage
2DCS
3DCS
CD34+
LIN-CD45RA- CD34+CD38low/-
LIN-CD45RA- CD34+CD38low/- CD49f+CD90+

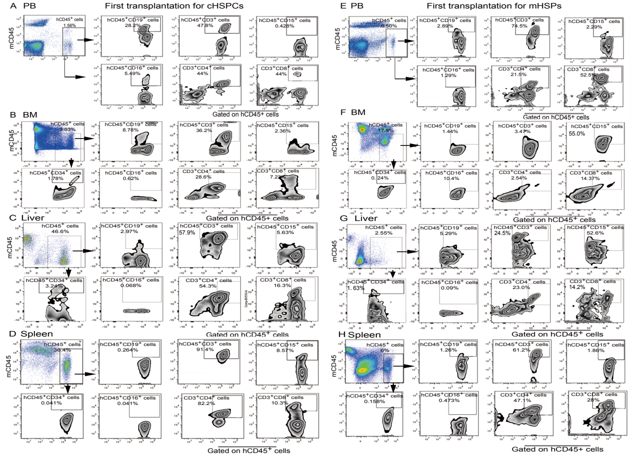
A PB
First transplantation for cHSPCs
E PB
First transplantation for mHSPs
B BM
F BM
C Liver
G Liver
D Spleen
H Spleen
mCD45
Gated on hCD45+ cells

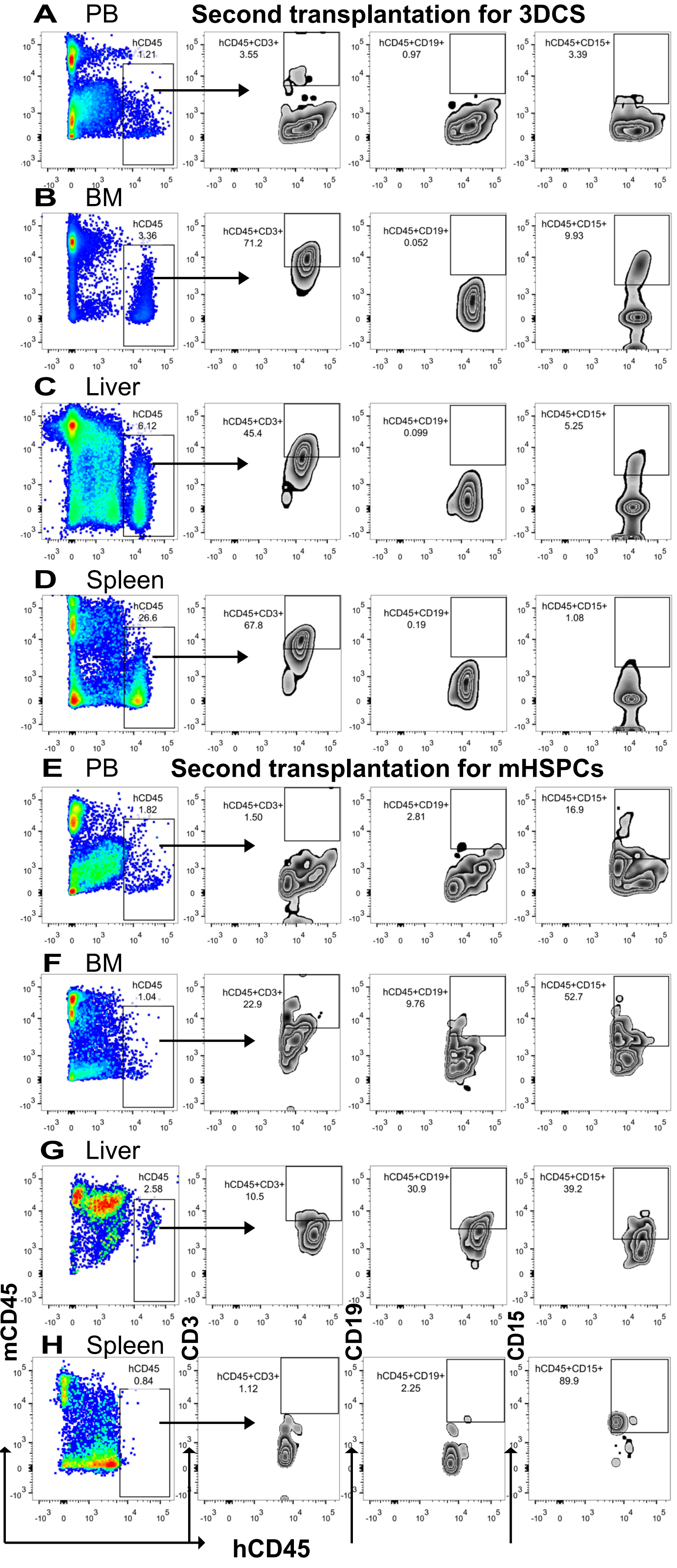
A PB
Second transplantation for 3DCS
hCD45 1.21
hCD45+CD3+ 3.55
hCD45+CD19+ 0.97
hCD45+CD15+ 3.39
B BM
hCD45 3.36
hCD45+CD3+ 71.2
hCD45+CD19+ 0.052
hCD45+CD15+ 9.93
C Liver
hCD45 6.12
hCD45+CD3+ 45.4
hCD45+CD19+ 0.099
hCD45+CD15+ 5.25
D Spleen
hCD45 26.6
hCD45+CD3+ 67.8
hCD45+CD19+ 0.19
hCD45+CD15+ 1.08
E PB
Second transplantation for mHSPCs
hCD45 1.82
hCD45+CD3+ 1.50
hCD45+CD19+ 2.81
hCD45+CD15+ 16.9
F BM
hCD45 1.04
hCD45+CD3+ 22.9
hCD45+CD19+ 9.76
hCD45+CD15+ 52.7
G Liver
hCD45 2.58
hCD45+CD3+ 10.5
hCD45+CD19+ 30.9
hCD45+CD15+ 39.2
H Spleen
hCD45 0.84
hCD45+CD3+ 1.12
hCD45+CD19+ 2.25
hCD45+CD15+ 89.9
mCD45
CD3
CD19
CD15
hCD45

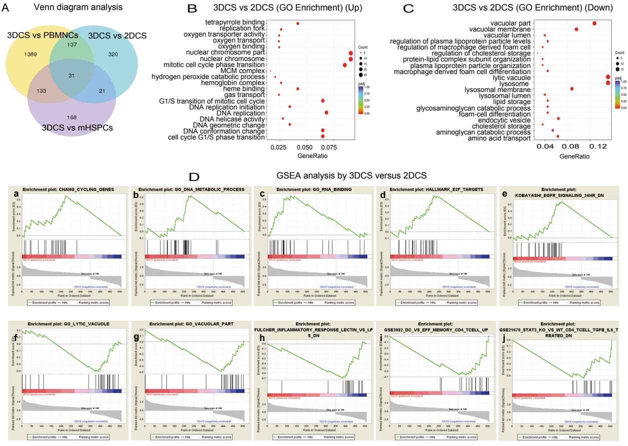
A
Venn diagram analysis
3DCS vs PBMNCs
3DCS vs 2DCS
3DCS vs mHSPCs
1389
137
320
31
133
21
168
B
3DCS vs 2DCS (GO Enrichment) (Up)
tetrapyrrole binding
replication fork
oxygen transporter activity
oxygen transport
oxygen binding
nuclear chromosome part
nuclear chromosome
mitotic cell cycle phase transition
MCM complex
hydrogen peroxide catabolic process
hemoglobin complex
heme binding
gas transport
G1/S transition of mitotic cell cycle
DNA replication initiation
DNA replication
DNA helicase activity
DNA geometric change
DNA conformation change
cell cycle G1/S phase transition
GeneRatio
Count
padj
C
3DCS vs 2DCS (GO Enrichment) (Down)
vacuolar part
vacuolar membrane
vacuolar lumen
regulation of plasma lipoprotein particle levels
regulation of macrophage derived foam cell
regulation of cholesterol storage
protein-lipid complex subunit organization
plasma lipoprotein particle organization
macrophage derived foam cell differentiation
lytic vacuole
lysosome
lysosomal membrane
lysosomal lumen
lipid storage
glycosaminoglycan catabolic process
foam-cell differentiation
endocytic vesicle
cholesterol storage
aminoglycan catabolic process
amino acid transport
GeneRatio
Count
padj
D
GSEA analysis by 3DCS versus 2DCS
a Enrichment plot: CHANG_CYCLING_GENES
b Enrichment plot: GO_DNA_METABOLIC_PROCESS
c Enrichment plot: GO_RNA_BINDING
d Enrichment plot: HALLMARK_E2F_TARGETS
e Enrichment plot: KOBAYASHI_EGFR_SIGNALING_24HR_DN
f Enrichment plot: GO_LYTIC_VACUOLE
g Enrichment plot: GO_VACUOLAR_PART
h Enrichment plot: FULCHER_INFLAMMATORY_RESPONSE_LECTIN_VS_LPS_DN
i Enrichment plot: GSE3982_DC_VS_EFF_MEMORY_CD4_TCELL_UP
j Enrichment plot: GSE21670_STAT3_KO_VS_WT_CD4_TCELL_TGFB_IL6_TREATED_DN
Enrichment score (ES)
Ranked list metric (Signal2Noise)
Rank in Ordered Dataset
Enrichment profile
Hits
Ranking metric scores

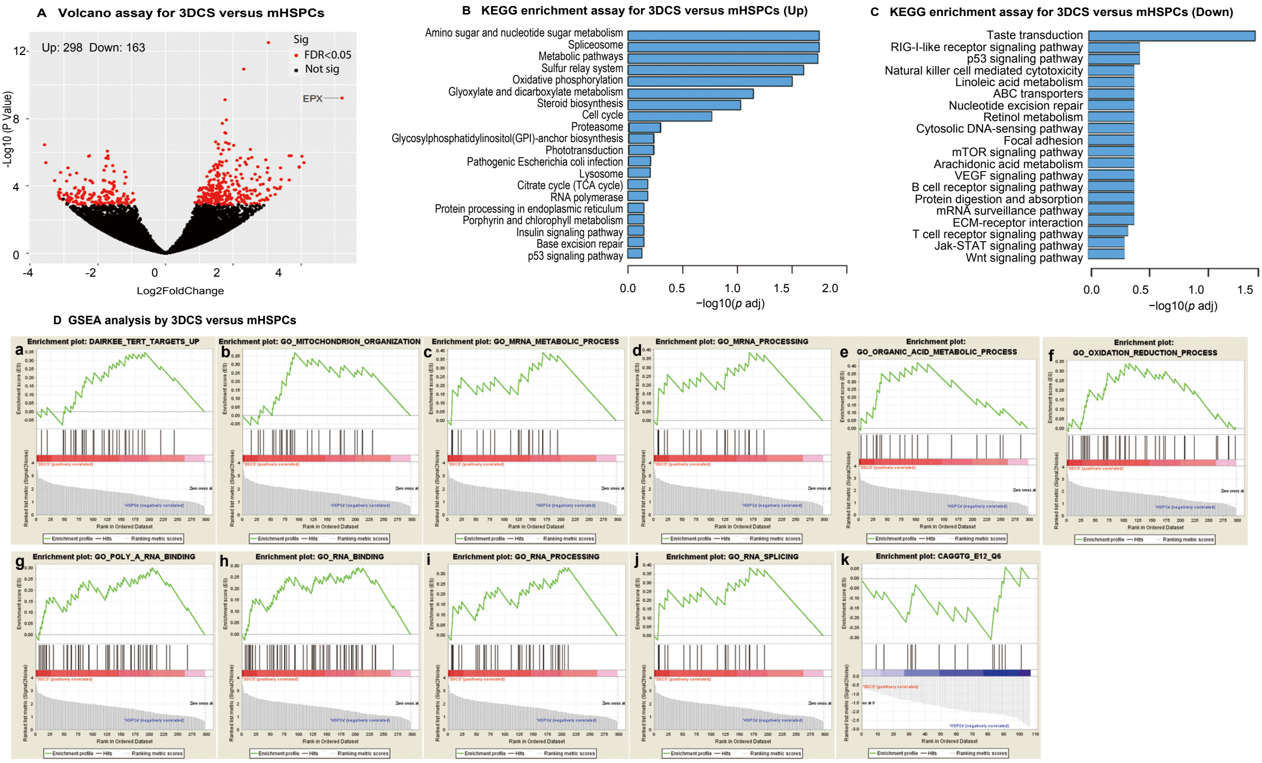
A Volcano assay for 3DCS versus mHSPCs
Up: 298 Down: 163
Sig
FDR<0.05
Not sig
EPX
-Log10 (P Value)
Log2FoldChange
B KEGG enrichment assay for 3DCS versus mHSPCs (Up)
Amino sugar and nucleotide sugar metabolism
Spliceosome
Metabolic pathways
Sulfur relay system
Oxidative phosphorylation
Glyoxylate and dicarboxylate metabolism
Steroid biosynthesis
Cell cycle
Proteasome
Glycosylphosphatidylinositol(GPI)-anchor biosynthesis
Phototransduction
Pathogenic Escherichia coli infection
Lysosome
Citrate cycle (TCA cycle)
RNA polymerase
Protein processing in endoplasmic reticulum
Porphyrin and chlorophyll metabolism
Insulin signaling pathway
Base excision repair
p53 signaling pathway
−log10(p adj)
C KEGG enrichment assay for 3DCS versus mHSPCs (Down)
Taste transduction
RIG-I-like receptor signaling pathway
p53 signaling pathway
Natural killer cell mediated cytotoxicity
Linoleic acid metabolism
ABC transporters
Nucleotide excision repair
Retinol metabolism
Cytosolic DNA-sensing pathway
Focal adhesion
mTOR signaling pathway
Arachidonic acid metabolism
VEGF signaling pathway
B cell receptor signaling pathway
Protein digestion and absorption
mRNA surveillance pathway
ECM-receptor interaction
T cell receptor signaling pathway
Jak-STAT signaling pathway
Wnt signaling pathway
−log10(p adj)
D GSEA analysis by 3DCS versus mHSPCs
a Enrichment plot: DAIRKEE_TERT_TARGETS_UP
b Enrichment plot: GO_MITOCHONDRION_ORGANIZATION
c Enrichment plot: GO_MRNA_METABOLIC_PROCESS
d Enrichment plot: GO_MRNA_PROCESSING
e Enrichment plot: GO_ORGANIC_ACID_METABOLIC_PROCESS
f Enrichment plot: GO_OXIDATION_REDUCTION_PROCESS
g Enrichment plot: GO_POLY_A_RNA_BINDING
h Enrichment plot: GO_RNA_BINDING
i Enrichment plot: GO_RNA_PROCESSING
j Enrichment plot: GO_RNA_SPLICING
k Enrichment plot: CAGGTG_E12_Q6
Enrichment score (ES)
Ranked list metric (Signal2Noise)
Rank in Ordered Dataset
Enrichment profile — Hits — Ranking metric scores

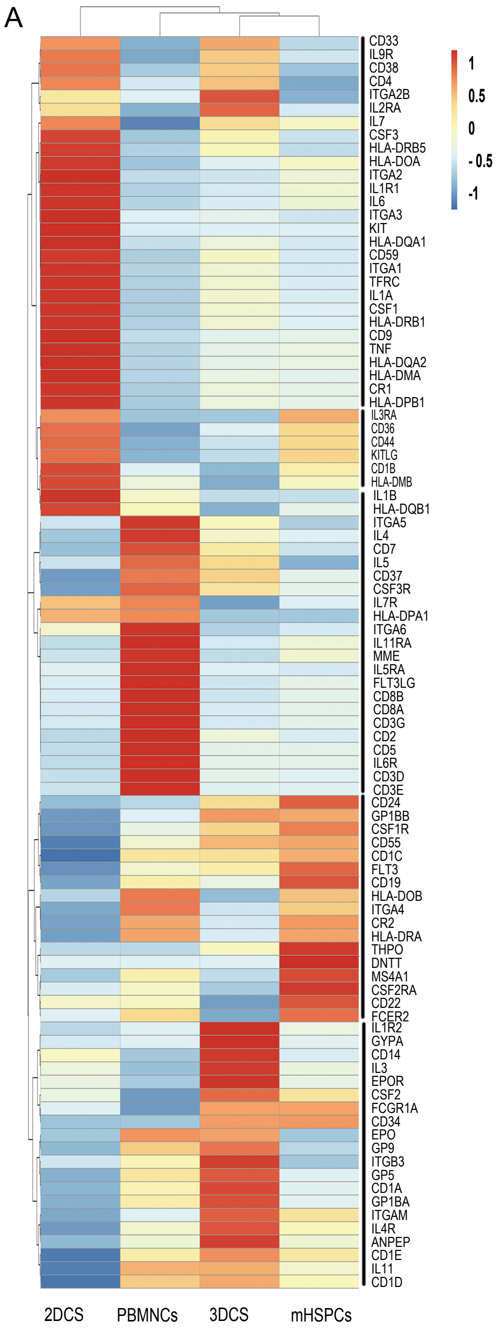
A
CD33
IL9R
CD38
CD4
ITGA2B
IL2RA
IL7
CSF3
HLA-DRB5
HLA-DOA
ITGA2
IL1R1
IL6
ITGA3
KIT
HLA-DQA1
CD59
ITGA1
TFRC
IL1A
CSF1
HLA-DRB1
CD9
TNF
HLA-DQA2
HLA-DMA
CR1
HLA-DPB1
IL3RA
CD36
CD44
KITLG
CD1B
HLA-DMB
IL1B
HLA-DQB1
ITGA5
IL4
CD7
IL5
CD37
CSF3R
IL7R
HLA-DPA1
ITGA6
IL11RA
MME
IL5RA
FLT3LG
CD8B
CD8A
CD3G
CD2
CD5
IL6R
CD3D
CD3E
CD24
GP1BB
CSF1R
CD55
CD1C
FLT3
CD19
HLA-DOB
ITGA4
CR2
HLA-DRA
THPO
DNTT
MS4A1
CSF2RA
CD22
FCER2
IL1R2
GYPA
CD14
IL3
EPOR
CSF2
FCGR1A
CD34
EPO
GP9
ITGB3
GP5
CD1A
GP1BA
ITGAM
IL4R
ANPEP
CD1E
IL11
CD1D
1
0.5
0
- 0.5
-1
2DCS
PBMNCs
3DCS
mHSPCs

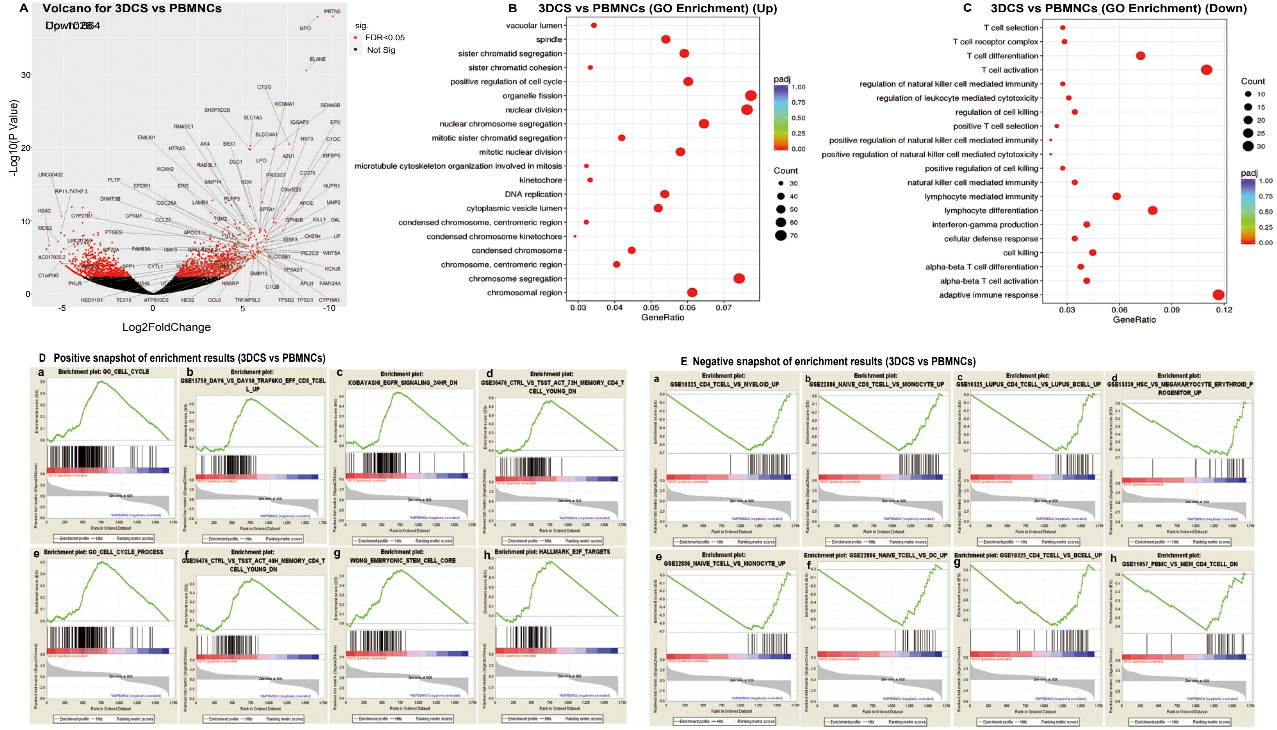
A Volcano for 3DCS vs PBMNCs
sig.
FDR<0.05
Not Sig
-Log10(P Value)
Log2FoldChange
B 3DCS vs PBMNCs (GO Enrichment) (Up)
vacuolar lumen
spindle
sister chromatid segregation
sister chromatid cohesion
positive regulation of cell cycle
organelle fission
nuclear division
nuclear chromosome segregation
mitotic sister chromatid segregation
mitotic nuclear division
microtubule cytoskeleton organization involved in mitosis
kinetochore
DNA replication
cytoplasmic vesicle lumen
condensed chromosome, centromeric region
condensed chromosome kinetochore
condensed chromosome
chromosome, centromeric region
chromosome segregation
chromosomal region
GeneRatio
padj
Count
C 3DCS vs PBMNCs (GO Enrichment) (Down)
T cell selection
T cell receptor complex
T cell differentiation
T cell activation
regulation of natural killer cell mediated immunity
regulation of leukocyte mediated cytotoxicity
regulation of cell killing
positive T cell selection
positive regulation of natural killer cell mediated immunity
positive regulation of natural killer cell mediated cytotoxicity
positive regulation of cell killing
natural killer cell mediated immunity
lymphocyte mediated immunity
lymphocyte differentiation
interferon-gamma production
cellular defense response
cell killing
alpha-beta T cell differentiation
alpha-beta T cell activation
adaptive immune response
GeneRatio
Count
padj
D Positive snapshot of enrichment results (3DCS vs PBMNCs)
a Enrichment plot: GO_CELL_CYCLE
b Enrichment plot: GSE15750_DAY6_VS_DAY10_TRAF6KO_EFF_CD8_TCELL_UP
c Enrichment plot: KOBAYASHI_EGFR_SIGNALING_24HR_DN
d Enrichment plot: GSE36476_CTRL_VS_TSST_ACT_72H_MEMORY_CD4_TCELL_YOUNG_DN
e Enrichment plot: GO_CELL_CYCLE_PROCESS
f Enrichment plot: GSE36476_CTRL_VS_TSST_ACT_40H_MEMORY_CD4_TCELL_YOUNG_DN
g Enrichment plot: WONG_EMBRYONIC_STEM_CELL_CORE
h Enrichment plot: HALLMARK_E2F_TARGETS
E Negative snapshot of enrichment results (3DCS vs PBMNCs)
a Enrichment plot: GSE10325_CD4_TCELL_VS_MYELOID_UP
b Enrichment plot: GSE22886_NAIVE_CD8_TCELL_VS_MONOCYTE_UP
c Enrichment plot: GSE10325_LUPUS_CD4_TCELL_VS_LUPUS_BCELL_UP
d Enrichment plot: GSE15330_HSC_VS_MEGAKARYOCYTE_ERYTHROID_PROGENITOR_UP
e Enrichment plot: GSE22886_NAIVE_TCELL_VS_MONOCYTE_UP
f Enrichment plot: GSE22886_NAIVE_TCELL_VS_DC_UP
g Enrichment plot: GSE10325_CD4_TCELL_VS_BCELL_UP
h Enrichment plot: GSE11057_PBMC_VS_MEM_CD4_TCELL_DN

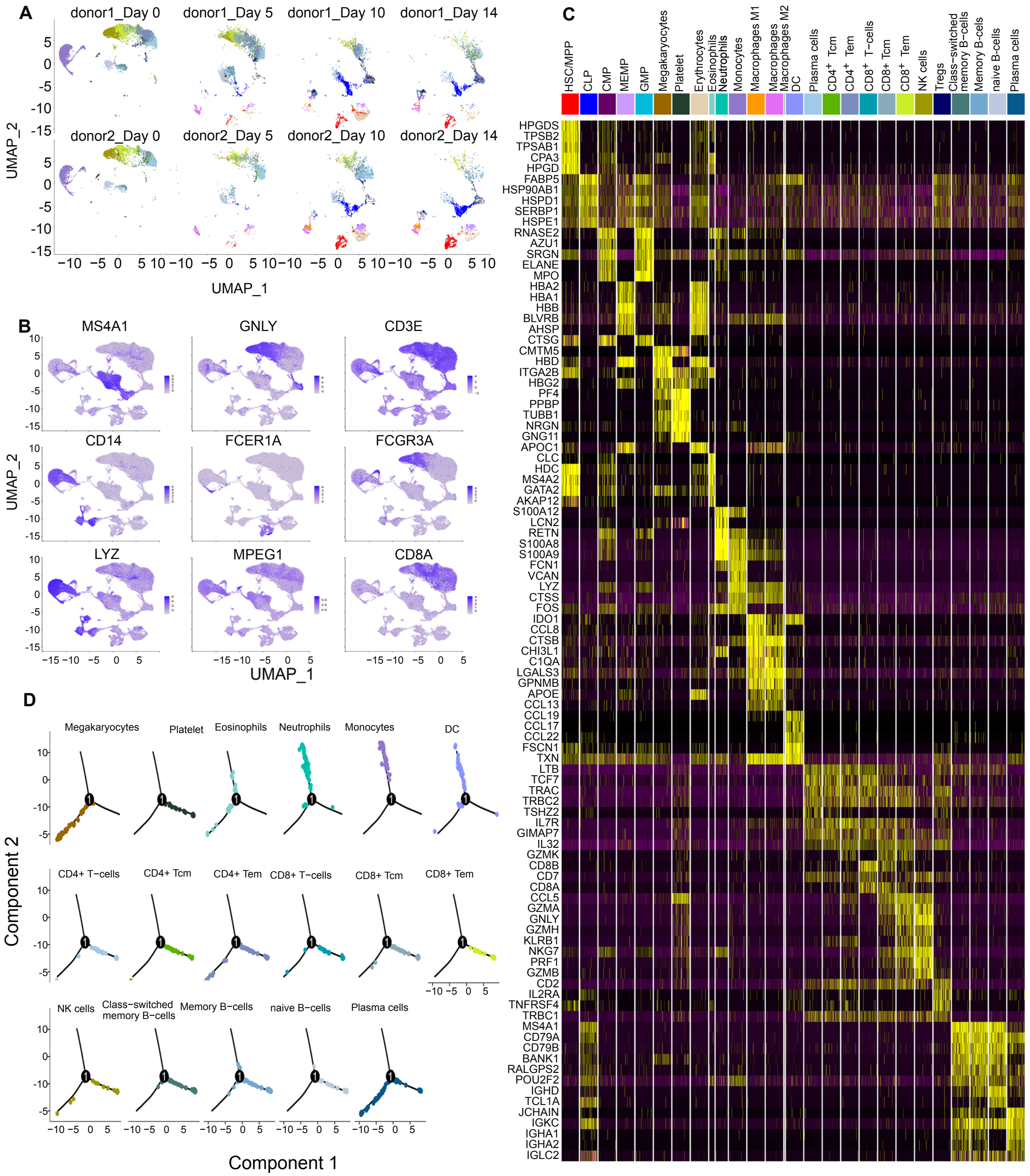

A
donor1_Day 0
donor1_Day 5
donor1_Day 10
donor1_Day 14
donor2_Day 0
donor2_Day 5
donor2_Day 10
donor2_Day 14
UMAP_2
UMAP_1
B
MS4A1
GNLY
CD3E
CD14
FCER1A
FCGR3A
LYZ
MPEG1
CD8A
C
HSC/MPP
CLP
CMP
MEMP
GMP
Megakaryocytes
Platelet
Erythrocytes
Eosinophils
Neutrophils
Monocytes
Macrophages M1
Macrophages
Macrophages M2
DC
Plasma cells
CD4+ Tcm
CD4+ Tem
CD8+ T-cells
CD8+ Tcm
CD8+ Tem
NK cells
Tregs
Class-switched memory B-cells
Memory B-cells
naive B-cells
Plasma cells
HPGDS TPSB2 TPSAB1 CPA3 HPGD FABP5 HSP90AB1 HSPD1 SERBP1 HSPE1 RNASE2 AZU1 SRGN ELANE MPO HBA2 HBA1 HBB BLVRB AHSP CTSG CMTM5 HBD ITGA2B HBG2 PF4 PPBP TUBB1 NRGN GNG11 APOC1 CLC HDC MS4A2 GATA2 AKAP12 S100A12 LCN2 RETN S100A8 S100A9 FCN1 VCAN LYZ CTSS FOS IDO1 CCL8 CTSB CHI3L1 C1QA LGALS3 GPNMB APOE CCL13 CCL19 CCL17 CCL22 FSCN1 TXN LTB TCF7 TRAC TRBC2 TSHZ2 IL7R GIMAP7 IL32 GZMK CD8B CD7 CD8A CCL5 GZMA GNLY GZMH KLRB1 NKG7 PRF1 GZMB CD2 IL2RA TNFRSF4 TRBC1 MS4A1 CD79A CD79B BANK1 RALGPS2 POU2F2 IGHD TCL1A JCHAIN IGKC IGHA1 IGHA2 IGLC2
D
Megakaryocytes
Platelet
Eosinophils
Neutrophils
Monocytes
DC
CD4+ T-cells
CD4+ Tcm
CD4+ Tem
CD8+ T-cells
CD8+ Tcm
CD8+ Tem
NK cells
Class-switched memory B-cells
Memory B-cells
naive B-cells
Plasma cells
Component 2
Component 1

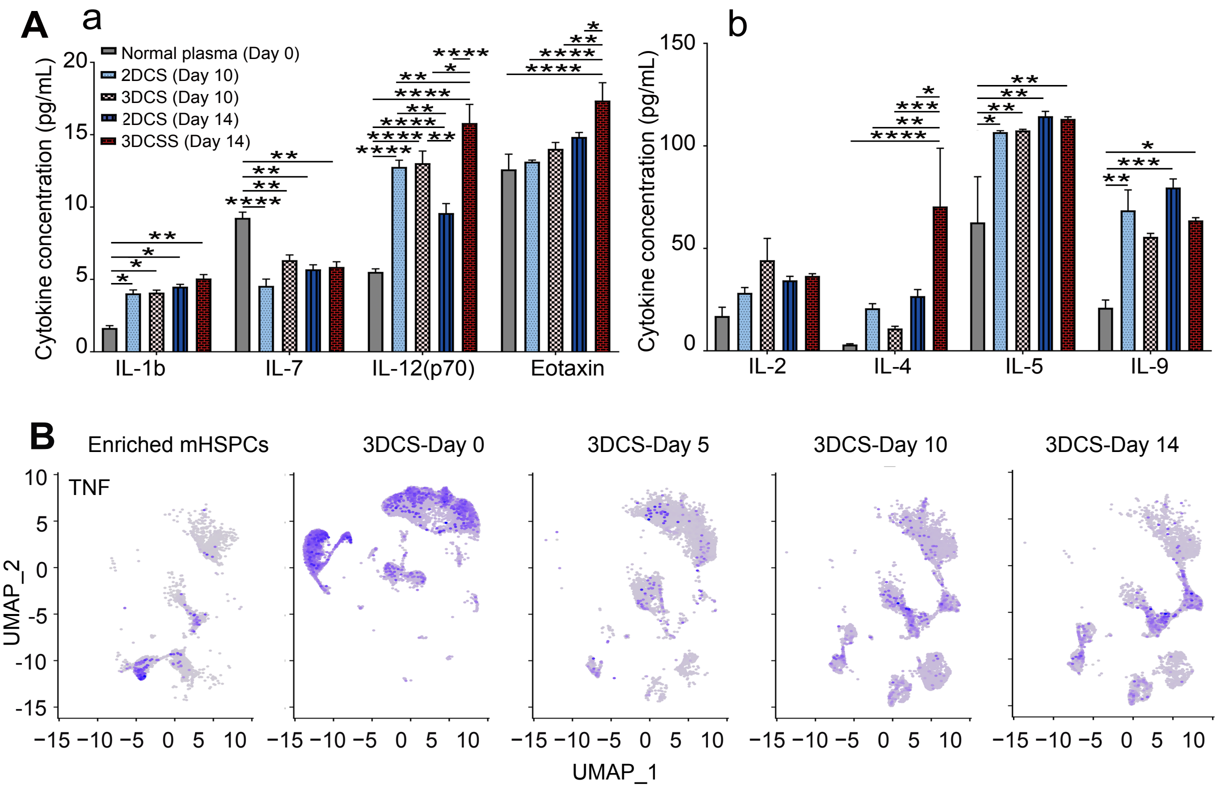

A
a
Normal plasma (Day 0)
2DCS (Day 10)
3DCS (Day 10)
2DCS (Day 14)
3DCSS (Day 14)
Cytokine concentration (pg/mL)
IL-1b
IL-7
IL-12(p70)
Eotaxin
b
Cytokine concentration (pg/mL)
IL-2
IL-4
IL-5
IL-9
B
Enriched mHSPCs
3DCS-Day 0
3DCS-Day 5
3DCS-Day 10
3DCS-Day 14
TNF
UMAP_2
UMAP_1
